# Bifurcation behaviors shape how continuous physical dynamics solves discrete Ising optimization

**DOI:** 10.1038/s41467-023-37695-3

**Published:** 2023-05-02

**Authors:** Juntao Wang, Daniel Ebler, K. Y. Michael Wong, David Shui Wing Hui, Jie Sun

**Affiliations:** 1 Theory Lab, Central Research Institute, 2012 Labs, Huawei Technologies Co. Ltd., Hong Kong SAR, China; 2 Department of Physics, Hong Kong University of Science and Technology, Hong Kong SAR, China

**Keywords:** Applied mathematics, Nonlinear phenomena

## Abstract

Simulating physical dynamics to solve hard combinatorial optimization has proven effective for medium- to large-scale problems. The dynamics of such systems is continuous, with no guarantee of finding optimal solutions of the original discrete problem. We investigate the open question of when simulated physical solvers solve discrete optimizations correctly, with a focus on coherent Ising machines (CIMs). Having established the existence of an exact mapping between CIM dynamics and discrete Ising optimization, we report two fundamentally distinct bifurcation behaviors of the Ising dynamics at the first bifurcation point: either all nodal states simultaneously deviate from zero (synchronized bifurcation) or undergo a cascade of such deviations (retarded bifurcation). For synchronized bifurcation, we prove that when the nodal states are uniformly bounded away from the origin, they contain sufficient information for exactly solving the Ising problem. When the exact mapping conditions are violated, subsequent bifurcations become necessary and often cause slow convergence. Inspired by those findings, we devise a trapping-and-correction (TAC) technique to accelerate dynamics-based Ising solvers, including CIMs and simulated bifurcation. TAC takes advantage of early bifurcated “trapped nodes” which maintain their sign throughout the Ising dynamics to reduce computation time effectively. Using problem instances from open benchmark and random Ising models, we validate the superior convergence and accuracy of TAC.

## Abstract (web summary)

Physical and physics-inspired computation is emerging as a new paradigm for tackling hard optimization problems. In this work, the authors establish rigorous mathematical conditions together with new design principles for physical as well as simulated dynamical systems to solve general Ising models.

## Introduction

Quadratic unconstrained binary optimization problems (QUBOs) are of great importance for a large variety of disciplines—encompassing systematic drug design^1^, simulation of molecular dynamics^2^, protein folding^3,4^, traffic flow optimization^5^, job scheduling^6^, and risk analyses in finance^7^, among others. Solving QUBOs was shown to be NP-hard^8^, limiting scalability of good solutions to the order of hundreds to thousands of variables—even for approximate algorithms^9–11^.

Alternative approaches based on the encoding of problems into the dynamics of physical systems have been proposed, drawing inspiration from the Hopfield and Hopfield-Tank neural networks^12–14^ from the 1980’s, with more general dynamics and control mechanisms, allowing for stronger scalability and convergence properties^15–22^. As experimental realizations of so-called Ising machines deem challenging^18,23–26^, the system dynamics have commonly been simulated on conventional computers^23,27–30^. Such approach has proven successful for medium- to large-scale optimizations of up to hundreds of thousand variables^19,28,31,32^, and even lead to special-purpose chip hardware implementations^33–37^.

Despite the reported performances, the simulated dynamics is accompanied by a fundamental issue: the equations of motion of Ising machines are continuous (real-valued), while the QUBO problem is discrete (binary). In particular, empirical observations as reported in refs. ^38,39^ suggested an intriguing interplay between nonlinearity of the dynamics (with a focus on coherent Ising machines (CIMs)) and structure of the coupling network in determining the success of solving the discrete Ising problem. Generally, relaxations of integers to reals often lead to suboptimal solutions^11,40–42^, due to a continuous interpolation of the discrete optimization landscape. Since the landscape changes dynamically during the evolution, it is essential to understand when Ising machines are able to identify correct solutions of the QUBO^38,39^. These insights would lead to lower bounds of the algorithm run-time, which can (1) prevent failure of the optimization because of too short evolutions, and (2) serve as a stopping indicator to avoid long execution times. Yet, such lower bounds have not been reported and are generally unknown.

In this work, we address the very question of when an Ising machine derived from continuous dynamics solves its corresponding discrete Ising model. Many of the findings are based on analysis of the simulated dynamics from the perspective of dynamical systems and bifurcation theory. We show that exact mapping between an Ising machine and the corresponding QUBO can only emerge when the control parameter of the evolution crosses a critical threshold. We prove sufficient conditions for exact solvability for the simulated dynamics, and attribute the conditions to a *synchronized bifurcation* of all nodal states. This scenario is found to impose lower bounds on the magnitudes of the nodal variables. Unlike previously reported, we show that individual nodal variables in Ising machines often do not bifurcate synchronously, but rather undergo a sequential cascade of instabilities. Such delayed bifurcation behavior constitutes a phenomenon which we refer to as *retarded bifurcation*. We attribute such retardations to the presence of *swing nodes*, which are subject to geometrical frustrations in the Ising machine. Swing nodes are found to oscillate in values around zero, potentially leading to unfeasible run-times and suboptimal solutions. Importantly, we show that the number of swing nodes in the state of the Ising machine at the first bifurcation point constitutes a key indicator for the deviation from the exact mapping threshold. Finally, we build upon these insights to design a trapping-and-correction (TAC) approach based on defrustration and stabilization mechanisms to accelerate convergence and enhance the performances of simulated physical solvers.

## Results

A newly emerging computational paradigm towards solving combinatorial optimization problems is based on integrating the problem into the dynamics of physical systems. Binary optimization problems can be represented as Ising Hamiltonians^43^,

1 $${H}_{{{{{{{{\rm{Ising}}}}}}}}}({{{{{{{\mathbf{\sigma }}}}}}}})=-\mathop{\sum}\limits_{i < j}{G}_{ij}{\sigma }_{i}{\sigma }_{j}=-\frac{1}{2}{{{{{{{{\mathbf{\sigma }}}}}}}}}^{T}G{{{{{{{\mathbf{\sigma }}}}}}}},$$

whose value is known as the Ising energy. Here **σ** ∈ {−1, +1}^*n*^ is the vector of *n* interacting spins *σ*_1_, *σ*_2_, . . . , *σ*_*n*_ and *G* represents a symmetric coupling matrix. Configurations σ^*^ which yield the minimum Ising energy are known as the ground states, and the corresponding *H*_Ising_(σ^*^) is the energy of the ground state. It was shown that many NP-complete and NP-hard problems can be cast in the form of minimizing *H*_Ising_(**σ**) in Eq. (1) over all possible binary configurations **σ**^44^.

So-called *Ising machines* integrate the Hamiltonian in Eq. (1) into a continuous dynamical system describing the temporal evolution of a network of coupled oscillators, such that by evolving towards its ground states, Ising machines simultaneously minimize *H*_Ising_. Different proposals of Ising machines include coherent Ising machines^16,45,46^ and quantum bifurcation machines^17,25^, among others^18,47–49^. The dynamics of a physical Ising machine is commonly simulated on conventional hardware. Generally, this leads to a characterization of the dynamics by a set of coupled differential equations as follows,

2 $${\left(\frac{d{{{{{{{\bf{x}}}}}}}}}{dt}\right)}_{i}={F}_{i}({{{{{{{\bf{x}}}}}}}},\,p)={f}_{i}({x}_{i},\,p)+\mathop{\sum}\limits_{j}{g}_{ij}({x}_{i},{x}_{j}),\quad i=1,\,2,{\ldots},\,n,$$

where ${{{{{{{\bf{x}}}}}}}}={({x}_{1},\ldots,{x}_{n})}^{T}\in {{\mathbb{R}}}^{n}$, *p* is a control parameter, and ${{{\bf{F}}}}({{{\bf{x}}}}, p)$ is the corresponding vector field. Here, the equations of motion originate from a continuous potential *U*(**x**, *p*) as − ∇ *U*(**x**, *p*) = **F**(**x**, *p*), and the functions *f*_*i*_ and *g*_*ij*_ implement the self and interactive couplings, respectively. This work focuses on coherent Ising machines (CIM) as a quantum-photonic implementation of physical Ising machines^16,27^, and we note that the derived results could be extended to other dynamics characterized by the general form of Eq. (2). For instance, in a CIM the state *x*_*i*_ corresponds to the in-phase amplitude of the *i*-th photon pulse whose dynamics is described by

3 $${f}_{i}(x,p)=(-1+p-{x}^{2})x,\,\, {g}_{ij}(x,y)=\xi {G}_{ij}y,$$

where *p* is the photon pump rate, *G*_*ij*_ is the coupling strength between pulses *i* and *j*, and *ξ* is a scaling factor. Typically the system is allowed to endure a transient period after which the continuous values *x*_*i*_ of the photon amplitudes are converted to the binary states *σ*_*i*_ = sign(*x*_*i*_). By setting *p* appropriately, it was shown that CIMs can successfully solve certain intermediate to large scale QUBOs^27^.

### Bifurcation analysis of CIM

**Fig. 1:**
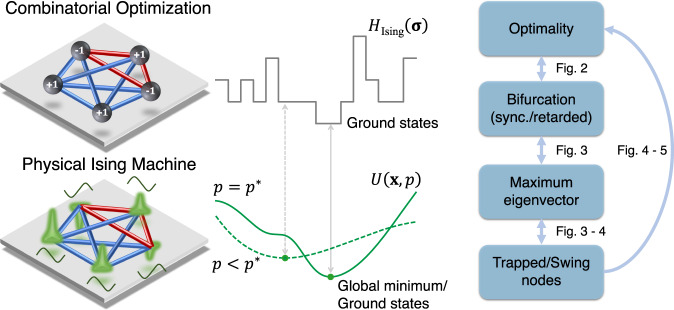
Mappings between the physical Ising machine and combinatorial optimization. The Ising machine solves a combinatorial optimization problem by embedding the discrete cost function into a physical model whose dynamics can be tuned by a control parameter *p*. At *p* = p^*^ (solid line), exact mapping happens as the global minimum of the embedded cost function (whose potential is denoted as *U*(**x**, *p*) herein) maps to that of the discrete problem whose cost function is denoted by *H*_Ising_(**σ**), leading to the correct solution. For *p* < p^*^ (dashed line), the mapping does not coincide, such that an optimization of the embedded cost function typically fails to find the correct solution. The relations between optimality of the solution found by the Ising machine, type of bifurcations exhibited by the system, properties of the system state at the first bifurcation point, and advantages through trapping-and-correction (TAC) approach presented in this work are illustrated in Figs. 2–5.

Given the continuous dynamics of CIMs, it is not clear whether its time evolution gives rise to the correct solutions of the discrete optimization problem. Recently, insightful perspectives of this problem were presented in ref. ^38^, shedding light on the intrinsic challenges caused by the complex topological structure of the phase space of the CIM dynamics and the interruptions in the adiabatic trajectories of fixed points. Success of the CIM strongly depends on the mapping between the continuous dynamics as in Eq. (2) and the discrete Hamiltonian *H*_Ising_, and it is believed that the minimum of the continuous potential *U*(**x**) can be mapped to the ground state of *H*_Ising_(**σ**) after binarization for certain control parameter values^50^ (see Fig. 1).

We investigate in the following when CIMs are fundamentally able to find a QUBO solution. Concretely, we show that when *p* is sufficiently large, i.e., $p \, > \,1+\frac{1}{2}\left(1+3\sqrt{3}\right){\max }_{l}{\sum }_{j}|\xi {G}_{lj}|$, there exists a stable state of the CIM dynamics whose binarization gives the ground state of the Ising problem (see Supplementary Note 1 for a proof). Then, we present a bifurcation analysis of the Ising dynamics. The results offer fundamental insights for practical optimization, as they provide mechanisms to determine the minimum run-time necessary for solving a QUBO. Bifurcations occur when the pump rate *p* is increased above certain thresholds *p*_*i*_ (*i* = 0, 1, . . . ), at which the qualitative dynamical behavior of the system is affected by instabilities^51^. Here, *p*_0_ denotes the first bifurcation point, *p*_1_ the second, and so on. Furthermore, we denote x^*^ as any fixed point of Eqs. (2, 3) which satisfies

4 $$(-1+p){{{{{{{{\bf{x}}}}}}}}}^{*}-{\left({x}_{1}^{*3},\ldots,{x}_{n}^{*3}\right)}^{T}+\xi G{{{{{{{{\bf{x}}}}}}}}}^{*}=0.$$

The evolution of the fixed points x^*^ with respect to the pump rate *p* can be derived as

5 $$\frac{d{{{{{{{{\bf{x}}}}}}}}}^{*}}{dp}={\left[(1-p)I+3{{{{{{{\rm{diag}}}}}}}}({x}_{1}^{*2},\ldots,{x}_{n}^{*2})-\xi G\right]}^{-1}{{{{{{{{\bf{x}}}}}}}}}^{*}.$$

For fixed *p*, the stability of the equilibrium point x^*^ is determined by the Jacobian matrix $J({{{{{{{{\bf{x}}}}}}}}}^{*},\,p) := \left[(-1+p)I-3{{{{{{{\rm{diag}}}}}}}}({x}_{1}^{*2},\ldots,{x}_{n}^{*2})+\xi G\right]$. Note that the trivial state **x**_0_ = **0** is always an equilibrium state (a fixed point) of the CIM. It is the unique solution until the first bifurcation point *p* = *p*_0_ at which the state **x**_0_ loses its stability, i.e., when the largest eigenvalue of $J({{{{{{{{\bf{x}}}}}}}}}_{0},\, p)=\left[(-1+p)I+\xi G\right]$ crosses zero. Thus the value *p*_0_ can be computed as ${p}_{0}=1-\xi {\lambda }_{\max }(G)$, where ${\lambda }_{\max }(G)$ is the largest eigenvalue of the coupling matrix *G*. The bifurcation direction $\frac{d{{{{{{{{\bf{x}}}}}}}}}_{0}}{dp}$ at **x**_0_ is the eigenvector of *J*(**x**_0_, *p*_0_) corresponding to the zero eigenvalue, denoted as ${{{{{{{{\bf{v}}}}}}}}}_{\max }$. We derive this result in more detail in Supplementary Note 2 and show that the corresponding equilibrium states (proportional to ${{{{{{{{\bf{v}}}}}}}}}_{\max }$) are indeed stable solutions. If the pump rate *p* is further increased, subsequent bifurcations may occur at (both stable and unstable) fixed points x^*^ if $J({{{{{{{{\bf{x}}}}}}}}}^{*},\, {p}^{{\prime} })$ has zero eigenvalue, and the bifurcation direction will be determined by the kernel of $J({{{{{{{{\bf{x}}}}}}}}}^{*},\, {p}^{{\prime} })$—here we use ${p}^{{\prime} }$ to denote the pump rate at a later bifurcation.

**Fig. 2:**
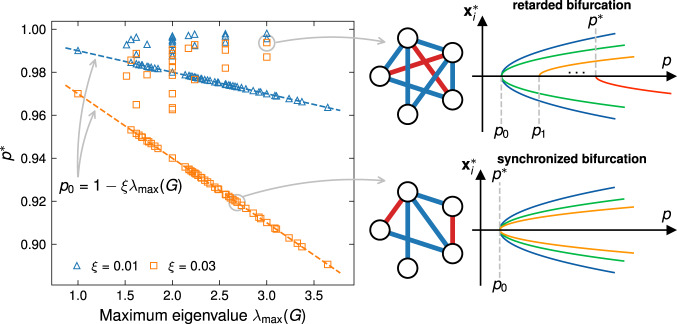
Retarded versus synchronized bifurcation. The point of exact mapping p^*^ vs. the largest eigenvalue of the interaction matrix *G* of 1000 graphs sampled randomly from all connected graphs with *n* = 5 nodes. Dashed lines represent the bifurcation thresholds ${p}_{0}=1-\xi {\lambda }_{\max }(G)$ where non-zero stable equilibrium first appears. While for the majority of the cases p^*^ = *p*_0_, outliers for which p^*^ > *p*_0_ do occur (shown as symbols deviating above the lines). Two problem instances, one with p^*^ = *p*_0_ and one with p^*^ > *p*_0_, are chosen and the problem structures and bifurcation dynamics of the corresponding system states x^*^ are shown, with red and blue couplings corresponding to *G*_*ij*_ = +1 and −1, respectively. For p^*^ = *p*_0_, all nodes bifurcate jointly (synchronized bifurcation). For p^*^ > *p*_0_, the graph of the outlier is subject to geometrical frustration—in this particular case two further bifurcations are necessary towards the solution (retarded bifurcation).

To elucidate the impact of the value of *p* on the ability of the Ising machine to find the correct ground state of *H*_Ising_, we consider random instances of connected graphs consisting of *n* = 5 variables with random symmetric coupling matrices *G* ∈ {+1, 0, −1}^*n*×*n*^. In the following, we use p^*^ to denote the minimum pump rate *p* under which there exists a local minimum x^*^ of *U*(**x**, *p*) whose binarization yields the ground state of the Ising problem (see Methods section for details of determinations of p^*^). Note that sometimes the system may not strictly undergo a bifurcation at p^*^ but instead some nodal states cross over to change its sign leading eventually to the optimal solution after binarization (see Supplementary Fig. 3). As shown in Fig. 2, for most instances, the pump rate p^*^ beyond which the CIM yields the *exact* ground state coincides with the first bifurcation point *p*_0_. On the other hand, there exist outliers with p^*^ > *p*_0_ for which higher pump rates are necessary to trigger further bifurcations to achieve exact mapping. Observations along this line have been presented in ref. ^39^ using Sherrington-Kirkpatrick models as a special class of Ising problems. The general working principle underlying such phenomena, however, has remained unclear—preventing constructive usage of the the bifurcation dynamics induced by non-linearity in the system. The finite and non-vanishing gap between the outliers and *p*_0_ decreases for smaller values of the coupling parameter *ξ* and increases otherwise. Note, however, that the gap does not vanish: for outliers, p^*^ > *p*_0_ will hold also for small ${\xi}$. Consequently, the need for outliers to further bifurcate towards exact mapping is robust against the choice of the coupling parameter. These insights offer a direct connection between the nature of bifurcations in the CIM and the optimality of the Ising machine for a given schedule.

**Fig. 3:**
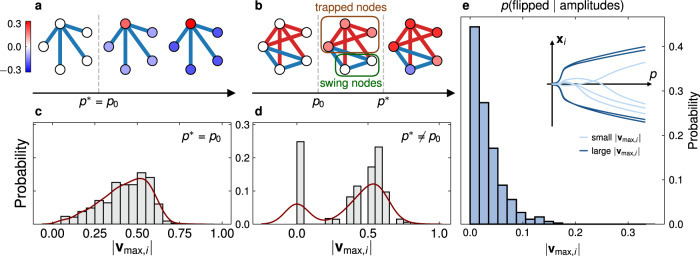
Trapped nodes and swing nodes that emerge during Ising dynamics and their impact on optimization. **a**, **b** Schematic illustrations of the evolution of the state corresponding to a global minimum point x^*^(*p*) following Eq. (5) in Ising problems for which (**a**) p^*^ = *p*_0_ (exact mapping arises right after first bifurcation), and (**b**) p^*^ > *p*_0_ (outlier). Outliers are accompanied by swing nodes, which bifurcate at higher pump rates (*p*_1_, . . . , p^*^) during the evolution (retarded bifurcations). In the corresponding graphs blue(red) edges denote *G*_*ij*_ = −1(+1), respectively. The nodes are colored according to the sign and magnitude of the steady-state components of the CIM dynamics. **c**, **d** Normalized counts of the magnitudes of the components of the maximum eigenvectors for models with (**c**) p^*^ = *p*_0_ and (**d**) p^*^ > *p*_0_, computed from statistics of 1000 random instances with *n* = 5 variables. The brown curves are the kernel density estimations of the probabilities. **e** The plot shows the probability that a variable with magnitude proportional to $|{{{{{{{{\bf{v}}}}}}}}}_{\max,i}|$ at the first bifurcation changes its sign during subsequent evolutions of the CIM, sampled from 100 fully connected graphs with *n* = 100 variables, and edge weights picked at random from a Gaussian distribution with zero mean and standard deviation $1/\sqrt{n}$. The inset (similar to Fig. 2) visualizes the evolution of several nodes in a typical 100-node graph with trapping and oscillations explicitly shown.

To understand the relation between the the system state ${{{{{{{{\bf{x}}}}}}}}}_{1}\propto {{{{{{{{\bf{v}}}}}}}}}_{\max }$ and the bifurcation type at *p*_0_, we analyze the evolution of the global minima states. Figure 3a, b shows the evolution of variable values of a problem instance with exact mapping at the first bifurcation point (p^*^ = *p*_0_), and of an outlier (p^*^ > *p*_0_) (for more details and further examples, see Supplementary Note 3). In the case p^*^ = *p*_0_, for the presented instance all variables of the CIM jointly drift away from zero after the first bifurcation and maintain their sign for growing values of *p*. We call such bifurcation synchronized. For outliers, some nodes remain close to or exactly zero unless further bifurcations occur. The need of the variable for further bifurcations at pump rates *p*_1_, *p*_2_, . . . is denoted as retarded bifurcation.

The empirical observations made in Fig. 3a, b guide us toward rigorous analytical results which we present in the following. Given the first non-trivial stable state ${{{{{{{{\bf{x}}}}}}}}}_{1}\propto {{{{{{{{\bf{v}}}}}}}}}_{\max }$, we quantify the degree of synchronization by

6 $${\alpha }^{2}({{{{{{{{\bf{x}}}}}}}}}_{1})={\left(\frac{{{{{{{{{\bf{x}}}}}}}}}_{1}^{T}}{||{{{{{{{{\bf{x}}}}}}}}}_{1}||}\frac{{{{{{{{{\mathbf{\sigma }}}}}}}}}_{1}}{||{{{{{{{{\mathbf{\sigma }}}}}}}}}_{1}||}\right)}^{2},$$

where we denote **σ**_1_ = sign(**x**_1_). Note that ${\alpha }^{2}({{{{{{{{\bf{x}}}}}}}}}_{1})={\alpha }^{2}({{{{{{{{\bf{v}}}}}}}}}_{\max })\in [0,\, 1]$ and if the components of **x**_1_ are more centralized away from zero, the larger the value of *α*^2^(**x**_1_). Then for any Ising Hamiltonian *H*(**σ**) (Eq. (1)), we have the following theorem.

#### Theorem 1

Given a non-zero real symmetric matrix *G* with diagonal entries equal to zero, defining an Ising Hamiltonian as in Eq. (1). Let ${{{{{{{{\bf{x}}}}}}}}}_{1}={({x}_{1,1},{x}_{1,2},\ldots,{x}_{1,n})}^{T}$ be the first non-trivial stable state of CIM (Eqs. (2, 3))—which is proportional to the maximum eigenvector of *G*, i.e., ${{{\bf{x}}}}_{1} \propto {{{\bf{v}}}}_{\max}$. Furthermore, denote Δ*H* = *H*_1_ − *H*_0_ as the energy gap between the energy *H*_1_ of the first excited state (here referred to as states/configurations that yield the second smallest Ising energy) and *H*_0_ of the ground state of *H*_Ising_(**σ**), and let ${\lambda }_{\max }$ and ${\lambda }_{\min }$ be the maximum and minimum eigenvalues of *G*. If

7 $${\alpha }^{2}({{{{{{{{\bf{x}}}}}}}}}_{1}) \, > \,{\alpha }_{c}^{2}({{{{{{{{\bf{x}}}}}}}}}_{1})=1-\frac{2\Delta H}{n({\lambda }_{\max }-{\lambda }_{\min })}$$

then **σ**_1_ = sign(**x**_1_) attains the energy of ground state, i.e.

8 $${H}_{{{{{{{{\rm{Ising}}}}}}}}}({{{{{{{{\mathbf{\sigma }}}}}}}}}_{1}) \, < \, {H}_{1}.$$

#### Proof

(A complete proof is presented in Supplementary Note 4.) □

Note that Theorem 1 implies two important findings: 1. if all nodes of a system exhibit a synchronized bifurcation—meaning that the magnitudes ∣*x*_1,*i*_∣, ∀ *i* = 1, 2, . . . , *n* of all nodes are sufficiently large (see Corollary 1 in Supplementary Note 4 and examples illustrated in Fig. 3c) at the first bifurcation point *p*_0_ such that the sufficient condition of Theorem 1 is satisfied—then the mapping between the continuous dynamics and the original optimization problem is exact. Further evolution of the CIM cannot improve the output. 2. If there exist nodes close to zero, leading to a distribution with a zero component in Fig. 3d, then the degree of synchronization is not large enough and the mapping requires further evolution beyond the first bifurcation to be exact.

Figure 3c, d shows the distribution of magnitudes $|{{{{{{{{\bf{v}}}}}}}}}_{\max,i}|$ of the components *i* = 1, 2, . . . , *n* of the maximum eigenvectors ${{{{{{{{\bf{v}}}}}}}}}_{\max }$ for 5000 random instances (*n* = 5 variables) with p^*^ = *p*_0_ and p^*^ > *p*_0_ respectively. See Supplementary Note 5 for additional instances. We discuss these two cases separately. (i) Instances with p^*^ = *p*_0_. It can be seen that no components are close to zero, corroborated by the observation that 88% of instances satisfy the sufficient condition in Theorem 1 and the average degree of synchronization is 〈*α*^2^(**x**_1_)〉 = 0.90, compared to the average threshold $\langle {\alpha }_{c}^{2}({{{{{{{{\bf{x}}}}}}}}}_{1})\rangle=0.80$. Hence the resulting state sign(**x**_1_) tends to yield the optimal solution to the problem. We also confirm experimentally (Fig. 3e) that the sign of nodes with large ∣*x*_1,*i*_∣ are not likely to change further with the increase of the pump rate *p*, and we call such nodes *trapped nodes*. (ii) Instances with p^*^ > *p*_0_. The distribution of magnitudes becomes bimodal: the components ∣*x*_1,*i*_∣ of the system state constitute an additional peak in counts for values around zero, with an average degree of synchronization 〈*α*^2^(**x**_1_)〉 = 0.73, compared to the average threshold $\langle {\alpha }_{c}^{2}({{{{{{{{\bf{x}}}}}}}}}_{1})\rangle=0.80$. Here, 92% of instances do not satisfy the sufficient condition in Theorem 1, and we observe that there is no full synchronization of all variable bifurcations. Note that there are still 8% of instances satisfying the condition yet they exhibit a retarded bifurcation, because although *H*(**σ**_1_) attains the ground state energy, there exists an index *j* such that ${({{{{{{{{\mathbf{\sigma }}}}}}}}}_{1})}_{j}={{{{{{{\rm{sign}}}}}}}}{({{{{{{{{\bf{x}}}}}}}}}_{1})}_{j}=0$. Hence, the binarization does not yield a valid Ising state. We also observe that if there exists at least one variable which does not bifurcate jointly after *p*_0_, then p^*^ > *p*_0_ and further bifurcations at larger values of *p* are necessary to fully determine the signs of those variables, c.f. Fig. 3b, d. We call such variables *swing nodes*, as they are observed to oscillate between negative and positive values around origin until a bifurcation traps them (Fig. 3e).

The occurrence of swing nodes indicates the presence of frustration in the system. Frustrations, which are known to generate multiplicity of local minima in combinatorial optimization, generally make the problem hard and cause high algorithmic complexity^52,53^. Consider the example in Fig. 3b. The two swing nodes are symmetric with respect to the permutation of their indices, i.e., (*z*_1_, *z*_2_) = (+, −) and (−, +) resulting in the same topological configuration. The degeneracy of these two states causes the stationarity of (0, 0) at the bifurcation point, and further increase of the pump rate is required to break the symmetry. Furthermore, we analyzed an additional 1000 random instances with *n* = 5 and found that whenever the first bifurcation state contains a zero component, it belongs to at least one frustrated loop. This constitutes a necessary condition for the occurrence of a swing node. We caution that the condition is necessary but not sufficient. Specifically, there are frustrated loops that do not result in swing nodes.

Generally speaking, as shown in Theorem 1, in the absence of retarded bifurcations, i.e., when degree of synchronization is sufficiently large at the first bifurcation, binarization of the maximum eigenvector yields the optimal solution. Since the maximum eigenvector can be computed to high numerical precision in polynomial time, those problems are computationally easy. On the other hand, when frustrations are present such that p^*^ > *p*_0_, it is generally difficult to classify the complexity of the problem, as the dynamics of the optimizer add an own layer of complexity to the problem^20^. The presence of retarded bifurcation and swing nodes become more significant with growing problem size *n*, leading to increasing deviations of the binarized first non-trivial state of the CIM from the optimal solution (see Supplementary Note 6). Consequently, instances typically get harder to solve with increasing size *n*. However, we observe that the ratio of swing nodes tends to saturate—thus enabling to utilize the trapped nodes when searching for optimal solutions.

### Trapping-and-correction (TAC) to boost the performance of Ising machines

For larger graphs, tracking the evolution of variable magnitudes across bifurcation points can be utilized as a key indicator for the mapping precision between global minima of the continuous potential and the discrete Ising ground states. Notice that such indicators also exist in other physics-inspired Ising machines, such as simulated bifurcation (SB) and its variants, including ballistic SB (bSB) and discrete SB (dSB)^19,28^. Since the exact mapping point p^*^ is generally unknown, the pump rate has to slowly increase until all magnitudes are *ϵ*-far away from zero. In practice, this poses three challenges: (i) Suboptimal solutions. Without exact knowledge of p^*^ there is no guarantee to stop the evolution precisely when the system is in the ground state. If *p* < p^*^, the fluctuations of swing nodes around zero assigns random values to the variables; for *p* > p^*^ the probability of finding the ground state by the CIM dynamics decreases with increasing *p* as more local minima emerge^16^; (ii) Long run-times. For retarded bifurcations in larger optimization problems many bifurcations might be needed to decide all swing nodes—often accompanied by unfeasible execution times; (iii) Insufficiency of the heuristics. With growing problem size, variables with small amplitudes are increasingly common (see Supplementary Note 6). In such scenarios, setting a good cut-off *ϵ* becomes more challenging as, in general, small amplitudes are also detected for instances with p^*^ = *p*_0_. Consequently, heuristic determination of the cut-off becomes unreliable.

**Fig. 4:**
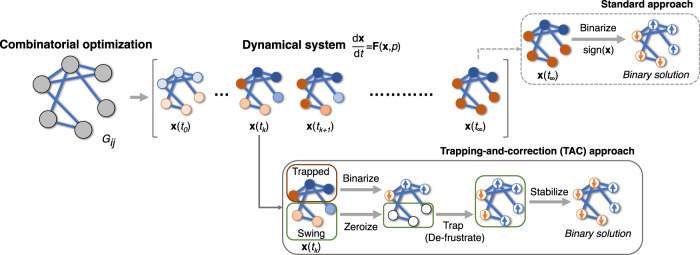
Schematic illustration of the trapping-and-correction (TAC) approach. It utilizes convergence properties of the early bifurcated “trapped nodes” to enable lazy updates and early stopping therefore effectively reduce computation time towards convergence of the Ising dynamics. See Supplementary Note 7 for details.

We now show how the above insights allow us to develop an acceleration technique which improves the speed of convergence when combined with dynamics-based Ising machines. This approach, which we term trapping-and-correction (TAC), is visualized in Fig. 4 and outlined in the next paragraph with a full description. Additional discussions are left to the Methods section and Supplementary Note 7. For notational simplicity, when used jointly with CIM dynamics, we call the resulting system TAC-CIM and similarly for other dynamics.

Here we use CIM and bSB^19^ (an improved version of SB which is robust and can achieve better results) to demonstrate how the TAC approach works. In TAC-CIM and TAC-bSB, the system is first evolved according to the dynamical equations in Eq. (3) and stopped at any arbitrary time *t*, with corresponding system state **x**(*t*). Then, nodes are classified into trapped and swing nodes based on a cut-off threshold $\epsilon=k||{{{{{{{\bf{x}}}}}}}}(t)||/\sqrt{n}$, with *k* being a tunable parameter. Trapped nodes, whose amplitudes are larger than the cut-off, are set to bit values according to their signs. Swing nodes, whose amplitudes are less than the cut-off, are randomly sequentially assigned binary values based on the interaction fields due to their neighbors. Then, every node is individually stabilized by examining the change in energy of the global state under bit flips. Note that this TAC approach is not restrictive to a particular form of dynamics, and can generally be used in other continuous-dynamics based Ising machines by simply adapting Eq. (2) while keeping the other parts the same.

**Fig. 5:**
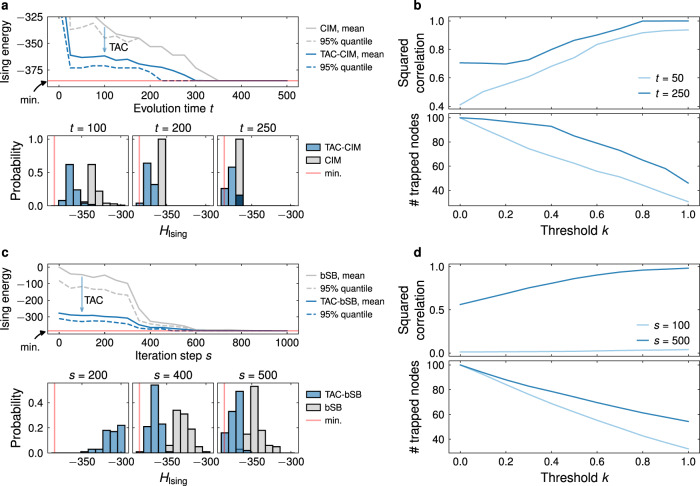
Accelerating dynamics-based Ising machines through the trapping-and-correction (TAC) technique. Comparison between Ising energies of (**a**) TAC-CIM and standard CIM, (**c**) TAC-bSB and standard bSB. The mean (solid curves) and the top 5% quantile (dashed curves) of Ising energies are illustrated at different times for a Max-cut problem with *n* = 100 variables (with respect to 100 trials, random in initialization and trapping order in TAC approach). The TAC-CIM and TAC-bSB result in stopping dynamics at the designated time and executing the TAC approach. The ground state is marked as “min” and depicted by solid straight lines. The bottom panel illustrates the probability of identifying the ground state for different times *t* during the evolution. For (**b**) CIM and (**d**) bSB, the number of trapped nodes with $|{x}_{i}(t)|\, > \, k||{{{{{{{\bf{x}}}}}}}}||(t)/\sqrt{n}$ and the squared correlation of their binarized values ${{{{{{{\rm{sign}}}}}}}}{({{{{{{{{\bf{x}}}}}}}}}_{i}(t))}_{i\in {V}_{{{{{{{{\rm{trapped}}}}}}}}}}$ with the corresponding ground state values ${({\sigma }_{i}^{{{{{{{{\rm{gs}}}}}}}}})}_{i\in {V}_{{{{{{{{\rm{trapped}}}}}}}}}}$ is shown as a function of the cut-off parameter *k* for different durations *t* of evolution. The simulation in (**a**) and (**c**) used *k* = 0.5 as the balance point between trapped and swing nodes.

Computational performance of the proposed TAC technique is shown in Fig. 5 for CIM and bSB. Here, we consider the Ising formulation of a Max-Cut problem g05_100.0 with *n* = 100 nodes taken from the benchmark dataset Biq Mac Library^54^. We refer to the Methods section for details of the simulation and Supplementary Note 8–9 for additional problem instances and comparisons with SB and dSB. Figure 5a, c illustrates the advantages of the TAC-CIM and TAC-bSB over standard CIM and bSB, for different evolution (stopping) times *t* of the dynamics. For standard CIM and bSB, Fig. 5b, d shows the size of the set of trapped nodes ${V}_{{{{{{{{\rm{trapped}}}}}}}}}=\{i:|{x}_{i}(t)|\, > \, k||{{{{{{{\bf{x}}}}}}}}(t)||/\sqrt{n}\}$, as well as the squared correlation between the binarized values ${{{{{{{\rm{sign}}}}}}}}{({{{{{{{{\bf{x}}}}}}}}}_{i}(t))}_{i\in {V}_{{{{{{{{\rm{trapped}}}}}}}}}}$ and the corresponding ground state values ${({{{{{{\sigma }}}}}}_{i}^{{{{{{{{\rm{gs}}}}}}}}})}_{i \in {V}_{{{{{{{{\rm{trapped}}}}}}}}}}$, as a function of the cut-off threshold *k* for different durations *t* of evolution. The correlation serves as an indicator for the correctness of classifying the nodes into trapped and swing. Small cut-offs lead to incorrect trapping of swing nodes. The resulting binarized values show low correlations to the ground state, and poor performance of the optimization. Large values of *k* lead to a small number of trapped nodes which have high correlation to the ground state. This can be understood from the low probability of nodes with large amplitudes to change signs after the first bifurcation point *p*_0_ (c.f. also Fig. 3e). However, large cut-offs lead to classifying a large number of nodes as swing, such that outputs are strongly decided by the stochastic stabilization. In this case too little information of the ground state configurations can be obtained from trapped nodes—which carry important features of the CIM and bSB evolution. For the simulations in this work the authors choose *k* = 0.5 to find approximately a balance between swing and trapped nodes. It can be seen that the proposed TAC approach significantly improves CIM and bSB with faster convergence and greater precision, by avoiding the need to perform full updates at every step of the dynamical evolution. For the presented instance, the top 5% of the 100 trials manage to identify the ground state in less than half of the run time needed by standard CIM and bSB, while for any stopping time TAC-CIM and TAC-bSB have advantages over standard CIM and bSB.

To further validate general applicability of the proposed TAC approach in accelerating convergence of algorithms beyond particular dynamics, we conducted a number of additional numerical experiments, to include most recent dynamics-based Ising machines such as SB, bSB and dSB^19,28^. The size of test graphs are increased to *n* = 2000 to examine possible performance variation in larger problem instances. Remarkably, in almost all examples and graph sizes the added TAC approach appears to be highly effective in accelerating convergence, regardless of the particular dynamical system (CIM, SB, or improved versions of SB—see Supplementary Note 9). This suggests the potential broad applicability of the proposed TAC approach to complement and generally improve the performance of dynamics-based Ising machines.

## Discussion

The emerging computational paradigm of utilizing continuous physical dynamics for solving combinatorial optimization problems proves to be a promising alternative to conventional algorithms. The ultimate success of this approach relies on understanding when evolving such continuous dynamics becomes equivalent to minimizing objectives with discrete variables. In this work we investigate this open question by analyzing the bifurcation dynamics of coherent Ising machines. We find that the Ising dynamics at the first bifurcation point fundamentally determines optimality of the continuous solution. At the first bifurcation, if all nodal states simultaneously change from zero to values that are *ϵ*-away from zero (synchronized bifurcation) we prove that the continuous dynamics solves the discrete optimization; on the other hand, when there is a partial set of nodes whose states remain *ϵ*-close to zero (retarded bifurcation) then further increase of the control parameter is necessary for the continuous system to evolve towards the desired discrete optimum. Such retardation in bifurcations constitutes a previously unnoted phenomenon, which correlates with the complexity of the problem and strongly impacts the performance of the Ising machine. We attribute retarded bifurcations to frustrations that are commonly inherent in the Hamiltonian, and necessitate the presence of small-amplitude components in the maximum eigenvector of the coupling matrix. The respective variables are called swing nodes whose values oscillate around the origin. Consequently, further bifurcations are necessary to determine the correct binary values of the nodes. In order to iteratively eliminate swing nodes, we develop a procedure that sequentially assigns binary values to swing nodes according to their local interactions in the network. The proposed trapping-and-correction (TAC) approach—drawing structural insights from CIM dynamics—was found to be effective in a number of dynamics-based Ising machines including CIM, SB, bSB and dSB, when extended to larger systems, yielding increased probability of finding ground states and reduced run-times. These results provide important insights regarding the link between continuous physical dynamics and discrete combinatorial optimization, and can potentially lead to enhanced parameter control schemes for nonlinear and physics-inspired optimization approaches.

## Methods

### Equivalence between QUBOs and Ising Hamiltonians

Given *n* binary variables ${\{{z}_{i}\}}_{i=1}^{n}\in {\{0,\, 1\}}^{n}$, a quadratic unconstrained binary optimization (QUBO) can be formulated mathematically as^55^

9 $$\mathop{\min }\limits_{{{{{{{{\bf{z}}}}}}}}\in {\{0,1\}}^{n}} q({{{{{{{\bf{z}}}}}}}})\,\mathop{=}\limits^{{{\rm{def}}}}\,\mathop{\min }\limits_{{{{{{{{\bf{z}}}}}}}}\in {\{0,1\}}^{n}} \mathop{\sum }\limits_{i,j=1;i\ne j}^{n}{Q}_{ij}{z}_{i}{z}_{j}+\mathop{\sum }\limits_{i=1}^{n}{Q}_{ii}{z}_{i}$$

where ${Q}_{ij}\in {\mathbb{R}}$. The objective function *q*(**z**) is minimized over all binary strings of length *n*, with a total of 2^*n*^ possible choices. Many NP-complete and NP-hard problems can be cast in the form of Eq. (9)^8,44,56,57^. Equivalently, a simple coordinate transformation *σ*_*i*_ ≔ 2*z*_*i*_ − 1 casts the objective function *q*(**z**) into an Ising Hamiltonian *H*_Ising_ of *n* interacting spins^43^

10 $${H}_{{{{{{{{\rm{Ising}}}}}}}}}({{{{{{{\mathbf{\sigma }}}}}}}})=-\mathop{\sum}\limits_{i < j}{G}_{ij}{\sigma }_{i}{\sigma }_{j}-\mathop{\sum }\limits_{i=1}^{n}{h}_{i}{\sigma }_{i}.$$

Here, *σ*_*i*_ ∈ {−1, +1} is the *i*-th spin value and coefficient *G*_*ij*_ denotes the coupling strength between spins *i* and *j*. The single-spin terms *h*_*i*_ can be absorbed by introducing one auxiliary spin *σ*_*n*+1_ and extending *G* by one row and one column to incorporate *h*_*i*_. Minimizing the energy of *H*_Ising_ then solves the corresponding QUBO. The explicit form of certain NP-complete problems as Ising Hamiltonians was derived in ref. ^44^.

### Determination of the exact mapping threshold

For the dynamics-based Ising machine Eq. (2), we define an exact mapping threshold p^*^ as the minimum *p* such that there exists a local minimum ${{{{{{{{\bf{x}}}}}}}}}_{p}^{*}$ of *U*(**x**, *p*) whose binarization leads to the ground state of the Ising problem. That is, let ${X}_{p}=\{{{{{{{{\bf{x}}}}}}}}|{{{{{{{\bf{F}}}}}}}}({{{{{{{\bf{x}}}}}}}},\, p)={{{\bf{0}}}}\,{{\mbox{and}}}\,{\lambda }_{\max }(J({{{{{{{\bf{x}}}}}}}},\, p)) \, < \, 0\}$ be the set of all stable equilibrium states under pump rate p and denote the ground state Ising energy as ${H}_{{{{{{{{\rm{Ising}}}}}}}}}^{*}={\min }_{{{{{{{{\mathbf{\sigma }}}}}}}} \in {\{-1,+1\}}^{n}}{H}_{{{{{{{{\rm{Ising}}}}}}}}}({{{{{{{\mathbf{\sigma }}}}}}}})$. Then ${p}^{*}={\inf }_{p}\{\exists {{{{{{{\bf{x}}}}}}}} \in {X}_{p},\, s.t.,\, {H}_{{{{{{{{\rm{Ising}}}}}}}}}({{{{{{{\rm{sign}}}}}}}}({{{{{{{\bf{x}}}}}}}}))={H}_{{{{{{{{\rm{Ising}}}}}}}}}^{*}\}$.

The exact value of p^*^ generally cannot be computed analytically, and requires numerical approximation methods. For the examples shown in this study, we manage to provide estimation for small-scale graphs, where the ground state Ising energy ${H}_{{{{{{{{\rm{Ising}}}}}}}}}^{*}$ can be computed through exhaustive search. Then, we use a bisection search to approximately locate p^*^, starting with an initial interval (*p*_*l*_, *p*_*r*_) where we typically choose *p*_*l*_ = *p*_0_ ≤ p^*^ and *p*_*r*_ be a sufficiently large number (to ensure that *p*_*r*_ > p^*^). Middle point of the interval *p*_*m*_ = (*p*_*l*_ + *p*_*r*_)/2 is examined, by using a large number of random states followed by Newton’s method to obtain an approximation ${\hat{X}}_{{p}_{m}}$ and then checking each element ${{{{{{{\bf{x}}}}}}}}\in {\hat{X}}_{{p}_{m}}$ for the equation ${H}_{{{{{{{{\rm{Ising}}}}}}}}}({{{{{{{\rm{sign}}}}}}}}({{{{{{{\bf{x}}}}}}}}))={H}_{{{{{{{{\rm{Ising}}}}}}}}}^{*}$. If for any element this holds then we conclude *p*_*m*_ ≥ p^*^ and update the right side of the interval *p*_*m*_ ← *p*_*r*_, otherwise we have *p*_*m*_ < p^*^ and we update the left side of the interval instead as *p*_*l*_ ← *p*_*r*_. This process continues until a pre-set resolution limit *ϵ* is met: ∣*p*_*l*_ − *p*_*r*_∣ < *ϵ*. The terminal value *p*_*m*_ is then used as an approximation of p^*^.

For the cases where only p^*^ = *p*_0_ or p^*^ > *p*_0_ needs to be decided, such as the example in Supplementary Note 5, we simply use the maximum eigenvector of *G* to determine ${\hat{X}}_{{p}_{0}}=\{{{{{{{{\bf{x}}}}}}}}|{{{{{{{\bf{x}}}}}}}}\propto {{{{{{{{\bf{v}}}}}}}}}_{\max }\}$ and check if ${H}_{{{{{{{{\rm{Ising}}}}}}}}}({{{{{{{\rm{sign}}}}}}}}({{{{{{{\bf{v}}}}}}}}))={H}_{{{{{{{{\rm{Ising}}}}}}}}}^{*}$ holds (if it holds, we conclude that p^*^ = *p*_0_, and otherwise p^*^ > *p*_0_).

### Simulation of the coherent Ising machines

The CIM is simulated by solving

11 $$\frac{d{x}_{i}}{dt}=\left(-1+p(t)\right) \, {x}_{i}-{x}_{i}^{3}+\xi \mathop{\sum}\limits_{j}{G}_{ij}{x}_{j},$$

using Euler method, the initial state is set as ${x}_{i}=A\cos {\theta }_{i}$, where *A* = 10^−3^ and *θ*_*i*_ is uniformed sampled from [0, 2*π*) and the Euler time step size is *dt* = 0.05. The parameters are set as $\xi=1/{\lambda }_{\max }(G)$, where ${\lambda }_{\max }(G)$ is the maximum eigenvalue of *G*, and the pump rate is linearly increased from the first bifurcation threshold to a value above 1, i.e., $p(t)={p}_{0}+\frac{t}{\tau }1.1\xi {\lambda }_{\max }(G)=1.1\frac{t}{\tau }$, where *τ* = 500 is the predefined maximum running time of the CIM.

### Simulation of the simulated bifurcation machine and its variants

The theory and dynamics of SB and its variants are summarized in refs. ^19, 28^, here we review the SB dynamics in the following for completeness. In particular, the SB dynamics is simulated by the modified explicit symplectic Euler method, and the iteration rules are given by^28^:

12 $${x}_{i}^{(m+1)}	={x}_{i}^{(m)}+\Lambda {y}_{i}^{(m)}\frac{\Delta t}{M},\\ {y}_{i}^{(m+1)}	={y}_{i}^{(m)}-\left[K{\left({x}_{i}^{(m+1)}\right)}^{3}+(\Lambda -p){x}_{i}^{(m+1)}\right]\frac{\Delta t}{M},\\ {x}_{i}({t}_{s+1})	={x}_{i}^{(M)}, {y}_{i}({t}_{s+1})={y}_{i}^{(M)}+\xi \mathop{\sum}\limits_{j}{G}_{ij}{x}_{j}^{(M)}\Delta t,$$

where *m* = 0, …, *M* − 1, *t*_*s*_ = *s*Δ*t*, *s* = 0, 1, …, *S*, $${x}_{i}^{(0)}={x}_{i}({t}_{s})$$, $${y}_{i}^{(0)}={y}_{i}({t}_{s})$$, and *p* = *p*(*t*_*s*+1_).

A variant of SB called bSB is simulated by the explicit symplectic Euler method with the following updating rules^19^:

13 $${y}_{i}({t}_{s+1})	={y}_{i}({t}_{s})+\left[(-\Lambda+p({t}_{s})){x}_{i}({t}_{s})+\xi \mathop{\sum}\limits_{j}{G}_{ij}{x}_{j}({t}_{s})\right]\Delta t,\\ {x}_{i}({t}_{s+1})	={x}_{i}({t}_{s})+\Lambda {y}_{i}({t}_{s+1})\Delta t,$$

and after the updates, if ∣*x*_*i*_∣ > 1, then *x*_*i*_ and *y*_*i*_ are set as sign(*x*_*i*_) and 0 respectively.

Another variant of SB is called dSB. Compared to bSB, the dSB discretizes the states in the couplings terms and its updating rules are as follows^19^

14 $${y}_{i}({t}_{s+1})	={y}_{i}({t}_{s})+\left[(-\Lambda+p({t}_{s})){x}_{i}({t}_{s})+\xi \mathop{\sum}\limits_{j}{G}_{ij}{{{{{{{\rm{sign}}}}}}}}({x}_{j}({t}_{s}))\right]\Delta t,\\ {x}_{i}({t}_{s+1})	={x}_{i}({t}_{s})+\Lambda {y}_{i}({t}_{s+1})\Delta t,$$

and after the updates, the same as bSB, if ∣*x*_*i*_∣ > 1, then *x*_*i*_ and *y*_*i*_ are set as sign(*x*_*i*_) and 0 respectively.

During the simulations, the settings of the parameters are as follows: For SB, *K* = 1 and *M* = 5; For SB, bSB, and dSB, Λ = 1, the time step Δ*t* = 0.5, the scaling factor ${\xi}=0.5 / \sqrt{\mathop{\sum}\limits_{ij} J^{2}_{ij} / (n - 1)}$, where *n* is the number of nodes, the scheduling of the pump rate $p({t}_{s})=\frac{s}{S}$, and the maximum iteration steps *S* = 1000. The initialization of the states are as follows: For SB, *x*_*i*_(0) = 0, and *y*_*i*_(0) is uniformly sampled from range [−0.1, 0.1]; For bSB and dSB, *x*_*i*_(0) and *y*_*i*_(0) are uniformed sampled from range [−0.1, 0.1].

### Trapping-and-correction approach

We propose a trapping-and-correction (TAC) approach which improves the performance of standard CIM and bSB both in convergence time and precision. The TAC approach consists of two main stages, defrustration and stabilization. In the following we explain the local stability criterion which is used for defrustration of the nodes, as well as stabilization of the system configuration. We consider the energy difference Δ*H*_*i*_ when flipping one spin *σ*_*i*_ ∈ {−1, 1} as ${\sigma }_{i} \, \mapsto \, {\sigma }_{i}^{{\prime} }=-{\sigma }_{i}$. Denoting by **σ**^(*i*)^ the spin vector for which the *i*-th spin is to be flipped, and by ${{{{{{{{\mathbf{\sigma }}}}}}}}}^{{(i)}^{{\prime} }}$ the resulting vector after the flip, the energy difference reads

15 $$\Delta {H}_{i}=H\left({{{{{{{{\mathbf{\sigma }}}}}}}}}^{{(i)}^{{\prime} }}\right)-H\left({{{{{{{{\mathbf{\sigma }}}}}}}}}^{(i)}\right)=({\sigma }_{i}-{\sigma }_{i}^{{\prime} })\mathop{\sum }\limits_{j=1,j\ne i}^{n}{G}_{ij}{\sigma }_{j}=2{\sigma }_{i}\mathop{\sum }\limits_{j=1,j\ne i}^{n}{G}_{ij}{\sigma }_{j},$$

where we have used the fact that the Ising problem can be written as ${H}_{{{{{{{{\rm{Ising}}}}}}}}}({{{{{{{\mathbf{\sigma }}}}}}}})=-\frac{1}{2}{{{{{{{{\mathbf{\sigma }}}}}}}}}^{T}G{{{{{{{\mathbf{\sigma }}}}}}}}$. Then, a configuration is stable if flipping a sign increases the energy, i.e., Δ*H*_*i*_ > 0, or equivalently

16 $$2{\sigma }_{i}\mathop{\sum }\limits_{j=1,j\ne i}^{n}{G}_{ij}{\sigma }_{j} \, > \, 0.$$

From Eq. (16) it follows directly that a configuration is stable if the sign(*σ*_*i*_) = sign(*y*_*i*_), with ${y}_{i}=\mathop{\sum }\nolimits_{j=1,j\ne i}^{n}{G}_{ij}{\sigma }_{j}$ and provided that other spins are not destabilized consequently. The approach utilizes the fact that stability is likely to increase when each swing node *k* is set to the value sign(*y*_*k*_). The stability criterion is used both for the defrustration of swing nodes (by assigning binary values according to Eq. (16)), as well as the stabilization of the full configuration (see Supplementary Note 7).

## Supplementary information

Supplementary Information

## Data Availability

Two types of datasets are used in this work. The public data comes from the Biq-Mac library^54^ which is an open-source dataset and is available at https://biqmac.aau.at/biqmaclib.html. The synthetic data are generated from random graph models, with the method and parameters of generation fully described in the main text and Supplementary Information.

## Code availability

Methods and algorithms, including their complete steps and choice of parameters are fully described in the main text and Supplementary Information. Full set of codes that support the findings of this study are available from the corresponding authors upon request.
