# Supplementary material for: Bifurcation behaviors shape how continuous physical dynamics solves discrete Ising optimization
Source: Nat Commun. 2023 May 2;14:2510. doi: 10.1038/s41467-023-37695-3 (PMC10154334; doi:10.1038/s41467-023-37695-3)
Supplement: Supplementary file 1 — Supplementary Information [file 41467_2023_37695_MOESM1_ESM.pdf]

# Supplementary Information for "Bifurcation behaviors shape how continuous physical dynamics solves discrete Ising optimization"

Juntao Wang[1,2], Daniel Ebler[1], K. Y. Michael Wong[2], David Shui Wing Hui[1], and Jie Sun[1]

[1]Theory Lab, Central Research Institute, 2012 Labs, Huawei Technologies Co. Ltd., Hong Kong SAR, China
[2]Department of Physics, Hong Kong University of Science and Technology, Hong Kong SAR, China

## Supplementary Note 1 – Existence of the mapping

Consider an $n$-dimensional Ising problem with Hamiltonian

$$H(\boldsymbol{\sigma}) = -\frac{1}{2}\boldsymbol{\sigma}^T G \boldsymbol{\sigma}, \tag{1}$$

where $G$ is a symmetric interaction matrix with $G_{ii} = 0$ and $\boldsymbol{\sigma} \in \{-1, +1\}^n$. The corresponding coherent Ising machine (CIM) dynamics is given as

$$\frac{dx_i}{dt} = (-1+p)x_i - x_i^3 + \xi \sum_j G_{ij} x_j, \tag{2}$$

where $x_i \in \mathbb{R}$. In the following, we show that if $p > 1 + \frac{1}{2}\left(1 + 3\sqrt{3}\right) D$, where $D = \max_l \sum_j |\xi G_{lj}| = ||\xi G||_1$, then there exists a stable fixed point of CIM dynamics [Supplementary Equation (2)] whose binarization corresponds to the ground states of Ising problem [Supplementary Equation (1)].

*Lemma* 1. Consider the fixed point equation of the CIM dynamics [Supplementary Equation (2)],

$$x_i'^3 - (-1+p)x_i' - \sum_j \xi G_{ij} x_j' = 0, \tag{3}$$

for $i = 1, 2, \ldots, n$. Let the intervals $I_{-1}$, $I_0$, and $I_{+1}$ be

$$I_{-1} = \left[-\frac{1}{2}\left(\sqrt{3(-1+p) - D} + \sqrt{\frac{-1+p+D}{3}}\right), -\sqrt{\frac{-1+p+D}{3}}\right], \tag{4}$$

$$I_0 = \left[-\frac{1}{2}\left(\sqrt{3(-1+p) - D} - \sqrt{\frac{-1+p+D}{3}}\right), \frac{1}{2}\left(\sqrt{3(-1+p) - D} - \sqrt{\frac{-1+p+D}{3}}\right)\right], \tag{5}$$

$$I_{+1} = \left[\sqrt{\frac{-1+p+D}{3}}, \frac{1}{2}\left(\sqrt{3(-1+p) - D} + \sqrt{\frac{-1+p+D}{3}}\right)\right], \tag{6}$$

where $D = \max_l \sum_j |\xi G_{lj}|$. If $p > 1 + \frac{1}{2}\left(1 + 3\sqrt{3}\right) D$, then for any $(s_1, s_2, \ldots, s_n) \in \{-1, 0, +1\}^n$, there exists exactly one $\mathbf{x}' \in I_{s_1} \times I_{s_2} \times \cdots \times I_{s_n}$ such that $\mathbf{x}'$ is a solution of Supplementary Equation (3).

*Proof.* Consider a set of compact subsets of Euclidean space $\mathbb{R}^{2n}$:

$$R = \left\{d(s_1, s_2, \ldots, s_n) = I_{s_1} \times I_{s_2} \times \cdots \times I_{s_n} \times I_a^n \mid s_i \in \{-1, 0, +1\}\right\}, \tag{7}$$

Daniel Ebler: ebler.daniel@huawei.com
Jie Sun: j.sun@huawei.com

where $I_a$ is defined as

$$I_a = \left[ -\frac{2(-1+p)-D}{3}\sqrt{\frac{-1+p+D}{3}}, \frac{2(-1+p)-D}{3}\sqrt{\frac{-1+p+D}{3}} \right]. \tag{8}$$

Then for $d(s_1, s_2, \ldots, s_n) \in R$, consider a mapping $F : d(s_1, s_2, \ldots, s_n) \to \mathbb{R}^{2n}$ such that $F(\mathbf{x}, \mathbf{y}) = (F_1(\mathbf{x}, \mathbf{y}), F_2(\mathbf{x}, \mathbf{y}), \ldots, F_{2n}(\mathbf{x}, \mathbf{y}))$ where $\mathbf{x} \in I_{s_1} \times I_{s_2} \times \cdots \times I_{s_n}$ and $\mathbf{y} \in I_a^n$, and

$$F_k(\mathbf{x}, \mathbf{y}) = \begin{cases} g_k^{-1}(y_k), & \text{if } k \le n, \\ \sum_j \xi G_{k-n,j} x_j, & \text{if } k > n, \end{cases} \tag{9}$$

where $g_k(x) = x^3 - (-1+p)x$ and $x \in I_{s_k}$. Note that it can be easily verified that $\forall s_k \in \{-1, 0, +1\}$, we have $g_k$ is monotonic in $I_{s_k}$ and $g_k : I_{s_k} \to I_a$ is bijective, so its inverse $g_k^{-1} : I_a \to I_{s_k}$ exists and is continuous. Then we can conclude that $F$ is a continuous mapping. Next we will show that if $p > 1 + \frac{1}{2}\left(1 + 3\sqrt{3}\right) D$, then (1) $d(s_1, s_2, \ldots, s_n) \cap d(s'_1, s'_2, \ldots, s'_n) = \emptyset$, if $(s_1, s_2, \ldots, s_n) \neq (s'_1, s'_2, \ldots, s'_n)$; (2) $\forall (s_1, s_2, \ldots, s_n) \in \{-1, 0, +1\}^n$, it follows that $F$ maps $d(s_1, s_2, \ldots, s_n)$ to itself.

For (1), it is valid by definition, as $I_{s_i} \cap I_{s'_i} = \emptyset$, for $s_i \neq s'_i$.

For (2), consider $(s_1, s_2, \ldots, s_n) \in \{-1, 0, +1\}^n$, and let $\mathbf{x} \in I_{s_1} \times I_{s_2} \times \cdots \times I_{s_n}$ and $\mathbf{y} \in I_a^n$. For $k = 1, 2, \ldots, n$, we have

$$F_k(\mathbf{x}, \mathbf{y}) = g_k^{-1}(y_k) \in I_{s_k}. \tag{10}$$

For $k = n+1, n+2, \ldots, 2n$, if $p > 1 + \frac{1}{2}\left(1 + 3\sqrt{3}\right) D$, we have

$$|F_k(\mathbf{x}, \mathbf{y})| = |\sum_j \xi G_{k-n,j} x_j| \le \sum_j |\xi G_{k-n,j}||x_j| \le \frac{D}{2}\left(\sqrt{3(-1+p)-D} + \sqrt{\frac{-1+p+D}{3}}\right) \tag{11}$$

$$= \frac{D}{2}\left(\sqrt{\frac{9(-1+p)-3D}{-1+p+D}} + 1\right)\sqrt{\frac{-1+p+D}{3}} \tag{12}$$

$$< \frac{2(-1+p)-D}{3}\sqrt{\frac{-1+p+D}{3}}, \tag{13}$$

and hence $F_k(\mathbf{x}, \mathbf{y}) \in I_a$, for $k = n+1, n+2, \ldots, 2n$. Thus we have shown that if $p > 1 + \frac{1}{2}\left(1 + 3\sqrt{3}\right) D$, then $F$ maps $d(s_1, s_2, \ldots, s_n)$ to itself.

Then by (1) and (2) and Brouwer's Fixed-Point Theorem [1], for any $d(s_1, s_2, \ldots, s_n) \in R$, there exists a fixed point $(\mathbf{x}', \mathbf{y}') \in d(s_1, s_2, \ldots, s_n)$ such that $F(\mathbf{x}', \mathbf{y}') = (\mathbf{x}', \mathbf{y}')$, or equivalently, $x_i'^3 - (-1+p)x'_i = \sum_j \xi G_{ij} x'_j$, for $i = 1, 2, \ldots, n$. As $|R| = 3^n$ by definition, we conclude that there are at least $3^n$ solutions of CIM dynamics Supplementary Equation (2). By the Finiteness Theorem (or specifically, by checking the condition on Gröner basis of the system of fixed points equation of Supplementary Equation (2)), this solution set of the system is zero-dimensional (as for each $i$, the Gröner basis has an element whose leading monomial is a power of $x_i$), hence by Bézout's Theorem [2], there are at most $3^n$ solutions. Therefore, we can further conclude that for any $(s_1, s_2, \ldots, s_n) \in \{-1, 0, +1\}^n$, there exists exactly one $x' \in I_{s_1} \times I_{s_2} \times \cdots \times I_{s_n}$ such that $x_i'^3 - (-1+p)x'_i - \sum_j \xi G_{ij} x'_j = 0$, for $i = 1, 2, \ldots, n$.

*Proposition* 1. If $p > 1 + \frac{1}{2}\left(1 + 3\sqrt{3}\right) D$, where $D = \max_l \sum_j |\xi G_{lj}|$, then there exists a stable fixed point of CIM dynamics (2) whose binarization corresponds to the ground states of Ising problem Supplementary Equation (1).

*Proof.* Let $\boldsymbol{\sigma}^{\text{gs}} = (\sigma_1^{\text{gs}}, \sigma_2^{\text{gs}}, \ldots, \sigma_n^{\text{gs}})$, $\sigma_i^{\text{gs}} \in \{-1, +1\}$, be the ground state of Ising problem Supplementary Equation (1). If $p > 1 + \frac{1}{2}\left(1 + 3\sqrt{3}\right) D$, then by Lemma 1, there exists exactly one $\mathbf{x}' \in I_{\sigma_1^{\text{gs}}} \times I_{\sigma_2^{\text{gs}}} \times \cdots \times I_{\sigma_n^{\text{gs}}}$ such that $\mathbf{x}'$ is the fixed point of the CIM dynamics Supplementary Equation (2). Since $\text{sign}(x'_i) = s_i$ for $x'_i \in I_{s_i}$ and $s_i \in \{-1, +1\}$, we can conclude that the binarization of $\mathbf{x}'$ corresponds the ground state, i.e., $\text{sign}(\mathbf{x}') = \boldsymbol{\sigma}^{\text{gs}}$.

Next, we show that $\mathbf{x}'$ is stable. The Jacobian matrix of the dynamics Supplementary Equation (2) at $\mathbf{x}'$ is given as

$$J|_{\mathbf{x}'} = \xi G + (-1+p)I + 3 \cdot \mathrm{diag}(-x_1'^2, -x_2'^2, \ldots, -x_n'^2). \tag{14}$$

By Courant-Fischer min-max Theorem and Lemma 1, its maximum eigenvalue $\lambda_{\max}(J|_{\mathbf{x}'})$ satisfies

$$\begin{aligned}
\lambda_{\max}(J|_{\mathbf{x}'}) &\leq \lambda_{\max}(\xi G) + \lambda_{\max}((-1+p)I) + \lambda_{\max}(3 \cdot \mathrm{diag}(-x_1'^2, -x_2'^2, \ldots, -x_n'^2)), && (15)\\
&\leq \lambda_{\max}(\xi G) + (-1+p) - 3 \cdot \min_i (x_i'^2), && (16)\\
&< \lambda_{\max}(\xi G) + (-1+p) - (-1+p+D) = \lambda_{\max}(\xi G) - D, && (17)
\end{aligned}$$

where $\lambda_{\max}(\cdot)$ is the maximum eigenvalue of the given matrix. Notice that

$$\lambda_{\max}(\xi G) \leq |\lambda_{\max}(\xi G)| \leq ||\xi G||_2 \leq ||\xi G||_1 = D, \tag{18}$$

and therefore we have

$$\lambda_{\max}(J|_{\mathbf{x}'}) < 0, \tag{19}$$

showing that $\mathbf{x}'$ is a stable fixed point.

## Supplementary Note 2 – Emergence of non-zero stable state at the first bifurcation

In this section, we derive the state of Coherent Ising Machine (CIM) after the first bifurcation point $p = p_0$ explicitly and prove its stability. The dynamical equations of the $n$-dimensional system $\mathbf{x} = (x_1, \ldots, x_n)^T$ are given as

$$\frac{dx_i}{dt} = (-1+p)x_i - x_i^3 + \xi \sum_j G_{ij} x_j, \tag{20}$$

and the corresponding potential function is

$$U(\mathbf{x}) = \frac{1}{4}\sum_i x_i^4 + \frac{(1-p)}{2}\sum_i x_i^2 - \frac{\xi}{2}\sum_{ij} x_i G_{ij} x_j, \tag{21}$$

For control parameter $p < p_0$ the variables assume the trivial state $\mathbf{x}_0 = \mathbf{0}$. When this state gets unstable, the system bifurcates into two separate branches, corresponding to the newly arising fixed points in Supplementary Equation (20). Depending on the problem, one or both of the branches can be stable, and generally (if no particular symmetries or structures are present) lead to different energy levels.

The initial state $\mathbf{x}_0$ gets unstable when the Jacobian $J|_{\mathbf{x}} = -\nabla^2 U(\mathbf{x})$ has positive maximum eigenvalue $\lambda_{\max}(J|_{\mathbf{x}})$. Explicitly, the Jacobian reads

$$J|_{\mathbf{x}} = (-1+p)I + \xi G - 3 \cdot \mathrm{diag}(x_1^2, ..., x_n^2) \tag{22}$$

and yields for the trivial state

$$J|_{\mathbf{x}_0} = (-1+p)I + \xi G \tag{23}$$

Analyzing the spectrum of $J|_{\mathbf{x}_0}$, we find the parameter $p_0$ as the minimum $p$ satisfying

$$0 \leq \lambda_{\max}(J|_{\mathbf{x}_0}) = -1 + p + \xi\lambda_{\max}(G) \tag{24}$$

Consequently, the trivial solution becomes unstable at $p_0 = 1 - \xi\lambda_{\max}(G)$ and the state $\mathbf{x}_0$ bifurcates into $\mathbf{x}_1 \propto \mathbf{v}_{\max}(G)$ (see main text).

Next, we show that $\mathbf{x}_1$ is a stable state. Stability at a point $\mathbf{x}$ is equivalent to the Jacobian $J|_{\mathbf{x}} = -\nabla^2 U(\mathbf{x})$ having positive maximum eigenvalue $\lambda_{\max}(J|_{\mathbf{x}})$. To perturb $J|_{\mathbf{v}_{\max}}$ around the bifurcation point $p_0$, we define $J|_{\mathbf{x}_1} = J|_{\mathbf{x}_0} + \delta J$, with $\delta J = -3\mathrm{diag}(\delta_1^2, ..., \delta_n^2)$ (see Supplementary Equation (22)). Then, we find [3]

$$\lambda_{\max}(J|_{\mathbf{x}_1}) \approx \lambda_{\max}(J|_{\mathbf{x}_0}) + \frac{v_{\max}^T \delta J v_{\max}}{v_{\max}^T v_{\max}} < 0 \tag{25}$$

following from $\lambda_{\max}(J|_{\mathbf{x}_0}) = 0$ and negative definiteness of $\delta J$. Hence, $\mathbf{x}_1$ corresponds to a stable local minimum.

In the following, we further derive the evolution of amplitudes of fixed points when $p$ is close to $p_0$. Given that the bifurcation direction of the CIM is proportional to the maximum eigenvector $\mathbf{v}_{\max}$ of the interaction matrix $G$, the stable CIM state at pump rate close to bifurcation point $p = p_0$ can be approximated by $\mathbf{x} = a\mathbf{v}_{\max}$. Then the parameter dependence of the amplitude of the fixed point $||\mathbf{x}||^2$ can be obtained as

$$\begin{aligned}\frac{d||\mathbf{x}||^2}{dp} = 2\mathbf{x}^T\frac{d\mathbf{x}}{dp} &= 2\xi\lambda_{\max}(G)\mathbf{x}^T\left(I - \frac{1}{\lambda_{\max}(G)}G + 3\mathrm{diag}(x_1^2, \ldots, x_n^2)\right)^{-1}\mathbf{x}, \\ &= 2\xi\lambda_{\max}(G)a^2\mathbf{v}_{\max}^T\left(K + 3a^2D^2\right)^{-1}\mathbf{v}_{\max},\end{aligned} \tag{26}$$

where we denote $K = I - \frac{1}{\lambda_{\max}(G)}G$ and $D = \mathrm{diag}(v_{\max,1}, \ldots, v_{\max,n})$. Note that $K$ is a singular matrix with kernel spanned by $\mathbf{v}_{\max}$. Here without loss of generality, we assume the dimension of the kernel is one. As $K$ is a symmetric matrix, its singular value decomposition

can be written as $K = USV^T$, where $U = (\mathbf{v}_{\max}, \mathbf{u}_1, \dots, \mathbf{u}_{n-1})$, $S = \mathrm{diag}(0, s_1, \dots, s_{n-1})$, and $V = (\mathbf{v}_{\max}, \mathbf{v}_1, \dots, \mathbf{v}_{n-1})$. Then Supplementary Equation (26) becomes

$$\begin{aligned}\frac{d||\mathbf{x}||^2}{dp} =& 2\xi\lambda_{\max}(G)a^2\mathbf{v}_{\max}^T\left(USV^T + 3UU^Ta^2D^2VV^T\right)^{-1}\mathbf{v}_{\max},\\ =& 2\xi\lambda_{\max}(G)a^2\mathbf{v}_{\max}^TU\left(S + 3a^2U^TD^2V\right)^{-1}V^T\mathbf{v}_{\max},\\ =& 2\xi\lambda_{\max}(G)a^2\mathbf{e}_1^T\left(S + 3a^2U^TD^2V\right)^{-1}\mathbf{e}_1,\\ =& 2\xi\lambda_{\max}(G)a^2\left[\left(S + 3a^2U^TD^2V\right)^{-1}\right]_{11},\end{aligned} \tag{27}$$

where $\mathbf{e}_1 = (1, 0, 0, \dots, 0)^T$. Notice that the $(i, j)$-th element of an inverse matrix $M_{ij}$ is given as

$$(M^{-1})_{ij} = \frac{\mathrm{cof}(M, j, i)}{\det(M)} = \frac{(-1)^{j+i}\det(M^{(j,i)})}{\det(M)} = \frac{\mathrm{cof}(M, j, i)}{\sum_k M_{1k}\mathrm{cof}(M, 1, k)}, \tag{28}$$

where $\mathrm{cof}(M, j, i)$ is the $(j, i)$ cofactor of matrix $M$ and $M^{(j,i)}$ is the $(n-1) \times (n-1)$ submatrix of $M$ by removing $j$-th row and $i$-th column. As the pump rate is close to bifurcation threshold, we have $a \to 0$, and $\mathrm{cof}(S + 3a^2U^TD^2V, i, j) = \mathrm{cof}(S, i, j)$, and therefore,

$$\left[\left(S + 3a^2U^TD^2V\right)^{-1}\right]_{11} = \frac{\mathrm{cof}(S, 1, 1)}{\sum_k (S + 3a^2U^TD^2V)_{1k}\mathrm{cof}(S, 1, k)}. \tag{29}$$

Notice that

$$\mathrm{cof}(S, 1, k) = \begin{cases}\prod_{i=1}^{n-1} s_i, & \text{if } k = 1\\ 0, & \text{otherwise}\end{cases} \tag{30}$$

so Supplementary Equation (29) can be simplified as

$$\begin{aligned}\left[\left(S + 3a^2U^TD^2V\right)^{-1}\right]_{11} =& \frac{\mathrm{cof}(S, 1, 1)}{(S + 3a^2U^TD^2V)_{11}\mathrm{cof}(S, 1, k)} = \frac{1}{(S + 3a^2U^TD^2V)_{11}},\\ =& \frac{1}{(3a^2U^TD^2V)_{11}} = \frac{1}{\mathbf{e}_1^T 3a^2U^TD^2V\mathbf{e}_1} = \frac{1}{3a^2\mathbf{v}_{\max}^TD^2\mathbf{v}_{\max}},\\ =& \frac{1}{3a^2\sum_i v_{\max,i}^4}.\end{aligned} \tag{31}$$

By plugging Supplementary Equation (31) into Supplementary Equation (27), we have

$$\frac{d||\mathbf{x}||^2}{dp} = \frac{2}{3}\frac{\xi\lambda_{\max}(G)}{\sum_i v_{\max,i}^4}. \tag{32}$$

## Supplementary Note 3 – Bifurcation analysis of the embedded potential

In this section we analyze the dynamics of the local minima points of the potential $U(\mathbf{x})$ during the bifurcation cascade of the system. This complements the discussion on the bifurcation behavior of the fixed points in the main text.

Supplementary Figure 1 shows the dynamics of a local minimum under increasing pump rate $p$ for an outlier with $n = 5$ variables. Until the first bifurcation threshold $p_0$ the value of the minima is constant at $U(\mathbf{x}_0)$. When reaching $p_0$ (solid grey line), some of the nodes bifurcate – leading to new emerging minima in $U$ – while others stay exactly zero. Hence, a mapping to the landscape of the discrete optimization problem is not possible at this point. Note that this is generally not the only possible scenario: all variables can be $\epsilon$-close to zero (swing nodes) after the bifurcation, but not exactly zero. In such a setting, the nodes have bifurcated but their magnitudes remain less than $\epsilon$ – which enables a mapping onto the QUBO landscape.

At $p = p^*$, further bifurcations have trapped all nodes. Stable minima emerging through the bifurcation cascade are drawn as solid lines in Supplementary Figure 1. The blue line corresponds to the minimum which is mapped to the global minimum of $H_{\text{Ising}}$. Green solid lines correspond to minima mapped to local minima (excited states) of $H_{\text{Ising}}$. Grey dashed lines show metastable branches of $U(\mathbf{x})$, meaning that they do not correspond to minima in $H_{\text{Ising}}$. Metastable states will further bifurcate with increasing values of $p$, resulting in other stable or metastable branches. In this way, more local minima emerge in the potential landscape of $U(\mathbf{x})$. Consequently, the probability to be in the global minima decreases for $p > p^*$.

Schematic illustrations of the evolutions of minima points for the case of Supplementary Figure 1 are shown in Supplementary Figure 2.

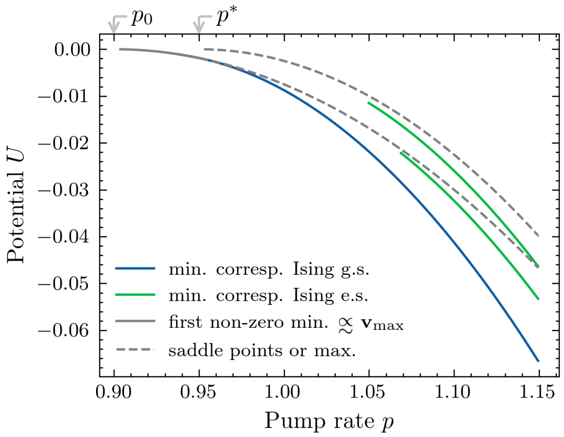


Supplementary Figure 1: Evolution of the potential $U$ of local minima points after the bifurcation in an Ising model with $n = 5$ variables. In this example, the decision point $p^* > p_0$ is larger than the bifurcation threshold, causing the problem to be an outlier.

Supplementary Figure 3 illustrates the same analysis for a larger system ($n = 20$). For the sake of visibility, only the evolution of the lowest minima of $U$ at each value $p$ is depicted. This example is slightly more involved. Different from the previous case of $n = 5$, the first bifurcation at $p = p_0$ causes all nodes to drift away from zero as shown in Supplementary Figure 3(b). In this instance, no node stays exactly zero. Consequently, the mapping of the minima of $U(\mathbf{x})$ between $p_0$ and $p^*$ onto the real problem is possible – in contrast to the case of $n = 5$ where some variables remained exactly zero. Nevertheless, variables $x_i$ with values close to zero indicate that they are swing nodes, since their small values result from cancellation of opposite fields of their neighbors. The binarization of their values leads to random assignments. This gives, ultimately, a mapping of $U(\mathbf{x})$ onto local minima of $H_{\text{Ising}}$ – hence, the green solid line in Supplementary Figure 3. For this example, the middle orange line (the most centric line around zero) represents state of a node whose value remains $\epsilon$-close to zero – no escape from the $\epsilon$- neighborhood around zero after $p_0$, but it is not until the pump rate reaches $p^*$ (marked by the vertical grey dashed line) that it escapes and crosses the zero line (we call it a crossover) and flip its sign from $-1$ to $+1$ leading to the exact

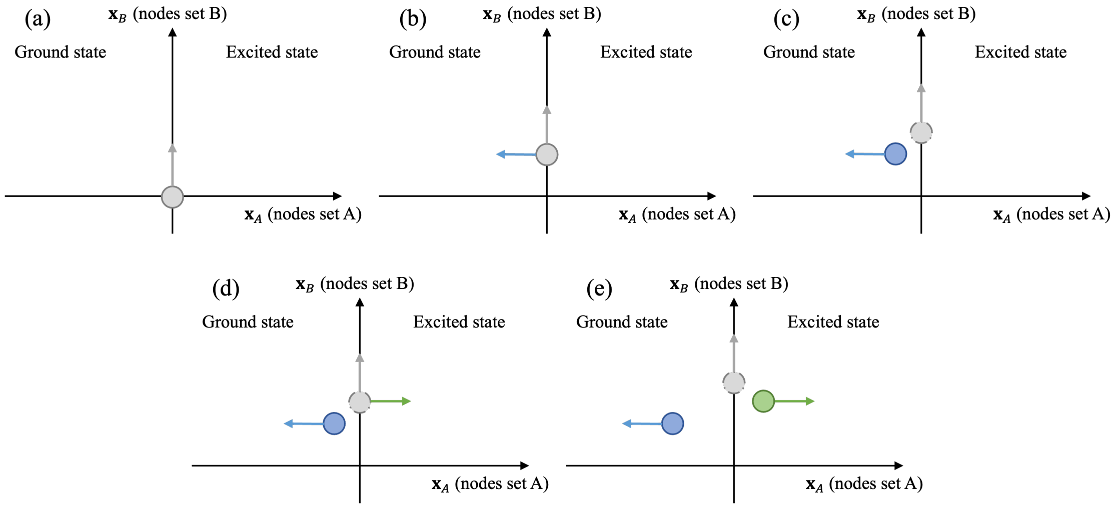


Supplementary Figure 2: A schematic illustration of the evolutions of the minima points in Supplementary Figure 1 as they approach the ground state and the first excited state of the corresponding Ising problem. The spaces that are mapped to the ground state and the first excited state are located in the upper left and right quadrants, respectively. (a) The bifurcation direction at $p = p_0$, at which some nodes decide their values (vertical axis) while other nodes remain swing nodes as their values are still exactly zero (horizontal axis). (b) The second bifurcation direction at some $p_1 > p_0$. (c) Relevant fixed points right after the second bifurcation: the dashed grey circle indicates the unstable fixed points that continue to evolve and the solid blue circle indicates the new emerged stable fixed point corresponding to the ground state of the Ising problem. (d) The third bifurcation at $p_2 > p_1$, where fixed points corresponding to metastable states of the Ising problem emerge from the unstable fixed points. (e) Fixed points right after the third bifurcation, where the green solid circle indicates the stable fixed point corresponding to the first excited state of the Ising problem.

mapping.

Until the point $p = p^*$, several further bifurcations and crossovers trap some of the nodes close to zero. For the potential $U(\mathbf{x})$, this means that new minima (stable and metastable) emerge, while existing ones evolve in the landscape (see Supplementary Figure 4). After the point $p = p^*$ is reached, all nodes are trapped or otherwise finished their crossover. One minimum in $U$ (solid blue line) evolves, with the corresponding state $\mathbf{x}^*$ mapped to the ground state of the the discrete problem.

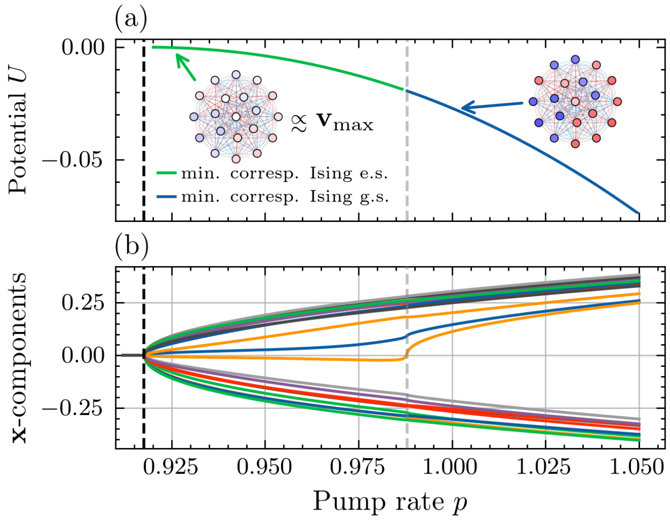


Supplementary Figure 3: Evolution of (a) the potential $U$ of local minima points. The node configurations corresponding to the Ising ground state and first excited state are shown. White nodes correspond to value zero. Red nodes stand for positive values and blue ones for negative values. The depth of the color indicates the magnitude of the variable assignment. (b) The components of a corresponding minimum point after the bifurcation in a fully-connected Ising model with $n = 20$ and random interactions $G_{ij} \in \{-1, +1\}$. The left dark dashed line indicates $p_0$ while the right grey dashed line indicates $p^*$.

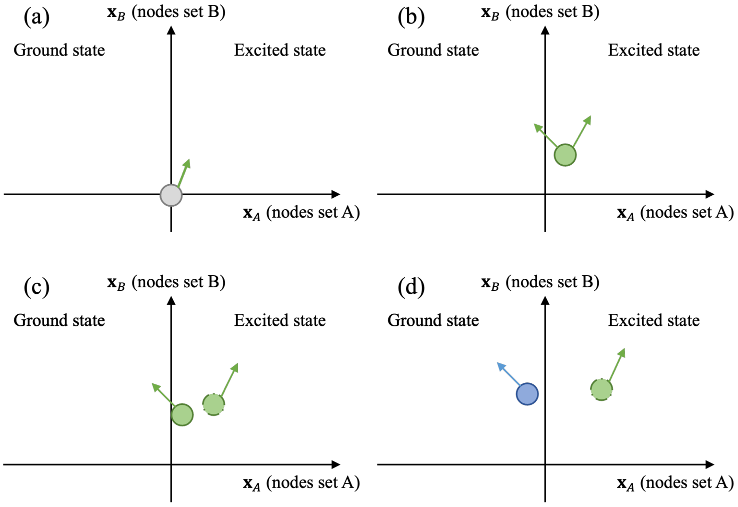


Supplementary Figure 4: A schematic illustration of the evolutions of the minima points in Supplementary Figure 3 as they approach the ground state and the first excited state of the corresponding Ising problem. The spaces that are mapped to the ground state and the first excited state are located in the upper left and right quadrants, respectively. (a) The bifurcation direction at $p = p_0$, at which some nodes get trapped (vertical axis) while other nodes remain close to zeros (horizontal axis). New emerging fixed point correspond to the first excited state. (b) The second bifurcation direction at $p_1 > p_0$. (c) Fixed points right after the second bifurcation, the dashed green circle indicates the evolution of unstable fixed points that continue to evolve and the solid green circle the new emerged stable fixed points. The latter still corresponds to the first excited states of the Ising problem. (d) Fixed points after further increase of $p$, where the stable fixed point evolves across the boundary and now corresponds to the ground state of the Ising problem (transit from solid green to solid blue).

## Supplementary Note 4 – Proofs of Theorems

In this section, we show that how the degree of synchronization of the first bifurcation in CIM implies the quality of solution after binarization and show that for large enough degree of synchronization, we can attain exact mapping at $p^* = p_0$ and the binarized maximum eigenvector $\text{sign}(\mathbf{v}_{\max})$ of $G$ corresponds to the optimal solution of $H(\boldsymbol{\sigma}) = -\frac{1}{2}\boldsymbol{\sigma}^T G \boldsymbol{\sigma}$.

Given the first non-zero stable state $\mathbf{x}_1 \propto \mathbf{v}_{\max}$, we can characterize the degree of synchronization by

$$\alpha^2(\mathbf{x}_1) = \left( \frac{\mathbf{x}_1^T}{||\mathbf{x}_1||} \frac{\boldsymbol{\sigma}_1}{||\boldsymbol{\sigma}_1||} \right)^2, \tag{33}$$

where we denote $\boldsymbol{\sigma}_1 = \text{sign}(\mathbf{x}_1)$. Note that $\alpha^2(\mathbf{x}_1) \in [0,1]$ and if the components of $\mathbf{x}_1$ are more centralized away from zero, the larger the value of $\alpha^2(\mathbf{x}_1)$. Then for any Ising problem, we have the following Theorem 1, which provides a sufficient condition for the optimality of $\boldsymbol{\sigma}_1$.

*Theorem* 1. Let $\mathbf{x}_1 = (x_{1,1}, x_{1,2}, \dots, x_{1,n})^T$ be the first non-trivial stable state of CIM – which is proportional to the maximum eigenvector of the interaction matrix defined in the Ising problem. Furthermore, denote $\Delta H = H_1 - H_0$ as the energy gap between the energy $H_1$ of the first excited state (here referred to as states/configurations that yield the second minimum Ising energy) and $H_0$ of the ground state of $H_{\text{Ising}}(\boldsymbol{\sigma})$, and let $\lambda_{\max}$ and $\lambda_{\min}$ be the maximum and minimum eigenvalues of $G$. If

$$\alpha^2(\mathbf{x}_1) > \alpha_c^2(\mathbf{x}_1) = 1 - \frac{2\Delta H}{n(\lambda_{\max} - \lambda_{\min})} \tag{34}$$

then $\boldsymbol{\sigma}_1 = \text{sign}(\mathbf{x}_1)$ attains the energy of ground state, i.e.

$$H_{\text{Ising}}(\boldsymbol{\sigma}_1) < H_1. \tag{35}$$

*Proof.* The main idea of the proof is to find a lower bound of the right hand side and a upper bound for the left hand side of the Supplementary Equation (35), then by showing that the upper bound is lower than the lower bound we prove the statement.

To find the lower bound of the right hand side, notice that we have the relaxation inequality

$$H_0 \geq \min_{||\mathbf{x}||^2 = n} H_{\text{Ising}}(\mathbf{x}) = -\frac{1}{2} n \lambda_{\max}, \tag{36}$$

and therefore

$$H_1 \geq -\frac{1}{2} n \lambda_{\max} + \Delta H. \tag{37}$$

Then to find a upper bound of the left hand side, notice that the Ising energy $H_{\text{Ising}}(\boldsymbol{\sigma}_1)$ can be written as

$$H_{\text{Ising}}(\boldsymbol{\sigma}_1) = -\frac{1}{2}\boldsymbol{\sigma}_1^T (G - \lambda_{\min} I)\boldsymbol{\sigma}_1 - \frac{n}{2}\lambda_{\min} = -\frac{1}{2}\sum_i (\lambda_i - \lambda_{\min})(\mathbf{v}_i^T \boldsymbol{\sigma}_1)^2 - \frac{n}{2}\lambda_{\min}, \tag{38}$$

as the interaction matrix can be eigen-decomposed as $G = \sum_i \lambda_i \mathbf{v}_i \mathbf{v}_i^T$, where $\mathbf{v}_i$ is the eigenvector of $G$ corresponding to eigenvalue $\lambda_i$. Notice that $\lambda_i - \lambda_{\min} \geq 0, \forall i$, we have

$$H_{\text{Ising}}(\boldsymbol{\sigma}_1) < -\frac{1}{2}(\lambda_{\max} - \lambda_{\min})(\mathbf{v}_{\max}^T \boldsymbol{\sigma}_1)^2 - \frac{n}{2}\lambda_{\min}. \tag{39}$$

Also as $\mathbf{x}_1 \propto \mathbf{v}_{\max}$, we have

$$\alpha^2(\mathbf{x}_1) = \left( \frac{\mathbf{x}_1^T}{||\mathbf{x}_1||} \frac{\boldsymbol{\sigma}_1}{||\boldsymbol{\sigma}_1||} \right)^2 = \frac{1}{n}(\mathbf{v}_{\max}^T \boldsymbol{\sigma}_1)^2, \tag{40}$$

and given the condition $\alpha^2(\mathbf{x}_1) > 1 - \frac{2\Delta H}{n(\lambda_{\max} - \lambda_{\min})}$, we have

$$H_{\text{Ising}}(\boldsymbol{\sigma}_1) < -\frac{n}{2}(\lambda_{\max} - \lambda_{\min}) + \Delta H - \frac{n}{2}\lambda_{\min} = -\frac{1}{2}n\lambda_{\max} + \Delta H \leq H_1, \tag{41}$$

which concludes the proof.

Then based on Theorem 1, we can further show that if all nodes of a system exhibit a synchronized bifurcation – meaning that the magnitudes $|x_{1,i}|, \forall i = 1, 2, ..., n$ of all nodes are sufficiently large at the first bifurcation point $p_0$, then the mapping between the continuous dynamics and the original optimization problem is exact.

*Corollary* 1. Suppose that the conditions stated in Theorem 1 hold, it follows that if

$$|x_{1,i}| \geq \epsilon = \sqrt{1 - \frac{2\Delta H}{n(\lambda_{\max} - \lambda_{\min})}} \frac{||\mathbf{x}_1||}{\sqrt{n}}, \tag{42}$$

then $\boldsymbol{\sigma}_1 = \text{sign}(\mathbf{x}_1) \in \{-1, +1\}^n$ is a ground state of the Ising problem.

*Proof.* If $|x_{1,i}| \geq \epsilon$, then we have

$$\alpha^2(\mathbf{x}_1) = \frac{1}{n||\mathbf{x}_1||^2} \left( \sum_i |x_{1,i}| \right)^2 \geq \frac{1}{n||\mathbf{x}_1||^2} n^2 \epsilon^2 \geq 1 - \frac{2\Delta H}{n(\lambda_{\max} - \lambda_{\min})}. \tag{43}$$

Therefore, by Theorem 1, $H_{\text{Ising}}(\boldsymbol{\sigma}_1)$ attains the energy of ground state, and hence $\boldsymbol{\sigma}_1 \in \{-1, +1\}^n$ is the ground state.

# Supplementary Note 5 – Retarded bifurcation detection

In this section we briefly describe the observations of retarded bifurcations. CIMs are commonly capable to decide a certain number of variables efficiently (at $p = p_0$). For such variables $i$, the magnitude $|x_i|$ is significantly larger than zero. Consequently, further evolution of the system (across additional bifurcation points) are not likely to change the sign of the variables anymore.

Variables $j$ for which $|x_j| < \epsilon$, with $\epsilon \ll 1$ can require longer (up to exponential) run-times to be trapped. These variables can be the cause of higher computational complexity of the respective optimization tasks. Hence, such retarded bifurcations are usually the cause for CIMs to output sub-optimal solutions, as the runs often do not reach $p = p^*$ or the CIM fails to stay at the state with the lowest potential during the evolution.

We exhaustively investigate the maximum eigenvectors of all possible instances with $n = 16$ of Ising problems on cubic graphs – i.e. graphs for which each node shares three links to other nodes (this set of graphs was previously studied, for instance, in Ref. [4]). The connections are $G_{ij} = -1$ so the Ising problems correspond to the max-cut problems on the cubic graphs. Supplementary Figure 5 shows the normalized counts of the magnitudes $|\mathbf{v}_{\max,i}|$ for all 4060 possible problems. It can be seen from Supplementary Figure 5(a) that for all problems with $p^* = p_0$, i.e., $\boldsymbol{\sigma} = \text{sign}(\mathbf{v}_{\max})$ is the ground state, the detected number of nodes close to zero is low. In contrast, problems subject to retarded bifurcations (with $p^* > p_0$) exhibit a proportionately dominant number of nodes close to zero – see Supplementary Figure 5(b). Hence, inspecting the largest eigenvector of the coupling matrix $G$ is a good indicator for the complexity of the problem.

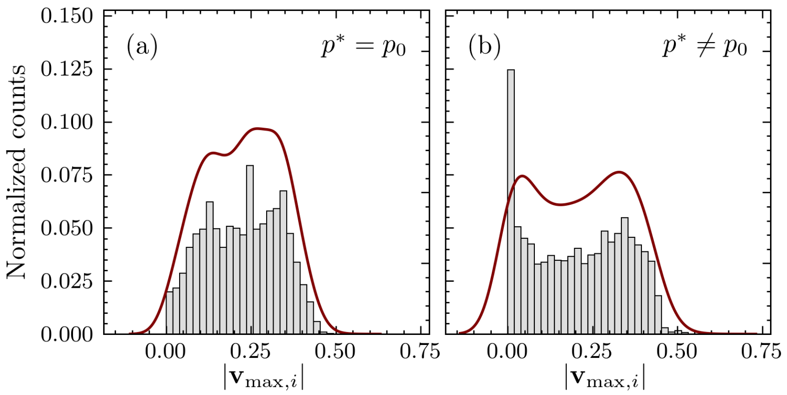


Supplementary Figure 5: Normalized counts of the magnitudes of the components $|\mathbf{v}_{\max,i}|$ of the normalized eigenvector $\mathbf{v}_{\max}$ for (a) models with $p^* = p_0$ and (b) models with $p^* > p_0$. Models are generated from all possible 4060 cubic graphs of order 16 with $G_{ij} = -1$ for all connections. The brown curves are the kernel density estimations of the probabilities.

## Supplementary Note 6 – Swing nodes and correctness of the solution at first bifurcation $p_0$

In this section we illustrate the statistics of the swing nodes and the degree of synchronization as a function of the size of graphs $n$ in Supplementary Figure 6. It can be seen that the presence of retarded bifurcation (low degree of synchronization) and swing nodes become more significant when $n$ increases, and they lead to increasing deviations (decreasing correctness) of the binary solution corresponding to the first non-trivial stable state of CIM from the correct solution (see Supplementary Figure 7).

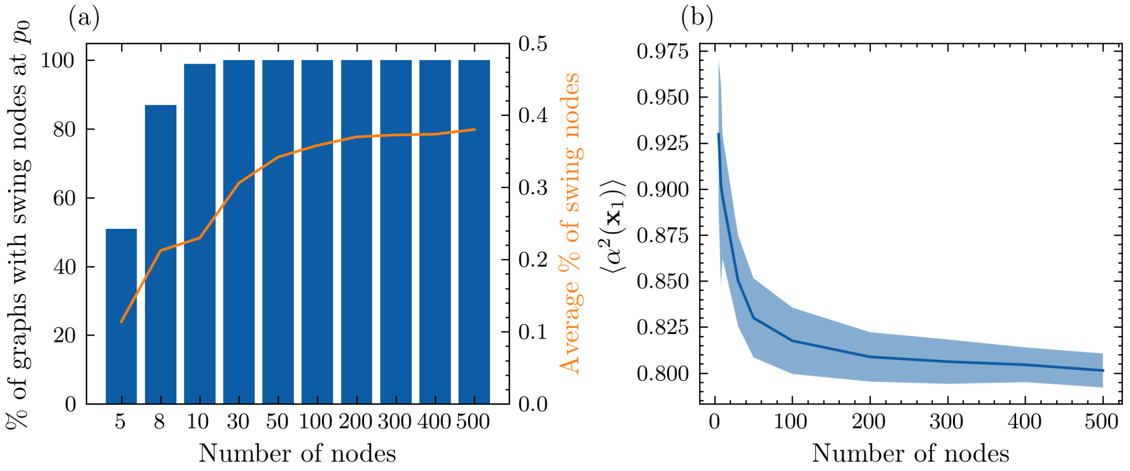


Supplementary Figure 6: Statistics of the swing nodes and the degree of synchronization for graphs with different number of nodes $n$. For each $n$, 100 fully connected graphs with random binary weights $(\pm 1)$ are generated to obtain the statistics. (a) The percentage of graph instances that contain at least one swing node at first bifurcation $p = p_0$ (blue bars) and the average percentage of swing nodes (orange curve) as a function of graph size $n$. Here the criterion for classifying nodes by first computing the state at the first bifurcation point as $\mathbf{x}_1 \propto \mathbf{v}_{\max}$, and then defining swing nodes as those $j$-th element such that $|\mathbf{x}_{1,j}| < \epsilon$, where the threshold is $\epsilon = 0.5||\mathbf{x}_1||/\sqrt{n}$. This figure shows that while the absolute number of swing nodes tend to increase with graph size, their percentage tends to saturate thus providing an opportunity to utilize the remainder (trapping nodes) when searching for an optimal solution. (b) Average degree of synchronization $\langle \alpha^2(\mathbf{x}_1) \rangle$ as a function of $n$, where the mean (solid curve) and one standard deviation (shaded area) are both shown. The saturation $\langle \alpha^2(\mathbf{x}_1) \rangle$ corresponds to the saturation of swing nodes fraction as in (a).

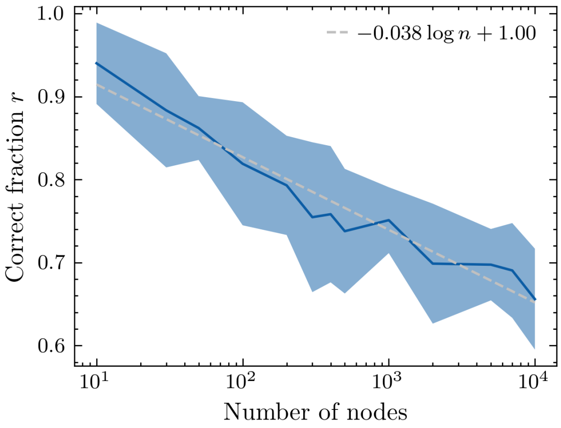


Supplementary Figure 7: The correct fraction $r$ using the binarized state at the first bifurcation $\mathbf{x}_1 \propto \mathbf{v}_{\text{max}}$ as the function of the graph size $n$. Let $\mathbf{s} = \text{sign}(\mathbf{x}_1) = \text{sign}(\mathbf{v}_{\text{max}})$ and denote the ground state configuration as $\boldsymbol{\sigma}^*$, the correct fraction is the fraction of binarized nodes agreeing with the ground state configuration, taken to be either $\boldsymbol{\sigma}^*$ or $-\boldsymbol{\sigma}^*$ due to the inversion symmetry of the objective function. It is computed as $r = \max\left(|\{i : \mathbf{s}_i = \boldsymbol{\sigma}_i^*\}|, |\{i : \mathbf{s}_i = -\boldsymbol{\sigma}_i^*\}|\right)$, where $|\cdot|$ represents cardinality of the set. For graphs with size $n > 10$, we estimate $\boldsymbol{\sigma}^*$ by running SB, bSB, as well as dSB each with 100 random initializations for an extended time and choosing the binary configuration that gave the lowest Ising energy. Average $r(n)$ is shown as the solid curve and shaded by one standard deviation, with a logarithmic fit denoted using the dashed line. In this figure, for each graph size $n$, a total of 20 fully connected graphs with random binary weights ($\pm 1$) are generated to obtain the statistics.

## Supplementary Note 7 – Trapping-and-correction (TAC) approach

In this section we define the trapping-and-correction (TAC) approach proposed in the main text and elaborate on the single steps in detail.

The approach takes a system state and a cutoff parameter $\epsilon = k||\mathbf{x}||/\sqrt{n}$ as inputs. The integer $k$ is fixed by the experimenter. Suppose the system dynamics stops at an instant $\hat{p}$ and given the state $\mathbf{x}_{\hat{p}}$ of the CIM or other dynamics-based Ising machine at $\hat{p}$, such as simulated bifurcation and its variants, the approach acts as follows.

**Algorithm:** trapping-and-correction (TAC) approach

```
Input: Variables indices V = {1, 2, ..., n} and state x_p̂
Output: Candidate binary solution σ of the Ising problem
// Identifying trapped nodes and swing nodes
for i ∈ V do
  | if |x_i(p̂)| < k||x_p̂||/√n then
  |   | σ_i ← 0
  | else
  |   | σ_i ← sign(x_i(p̂))
  | end
end
// The set of swing nodes
V_0 ← {i : σ_i = 0}
// Assign discrete values to swing nodes
while V_0 ≠ ∅ do
  | randomly pick i ∈ V_0
  | y_i ← Σ_j G_ij σ_j
  | σ_i ← sign(y_i)
  | V_0 ← V_0 \ i
end
// Stabilize the full configuration
while V ≠ ∅ do
  | randomly pick i ∈ V
  | y_i ← Σ_j G_ij σ_j
  | σ_i ← sign(y_i)
  | if σ_i = 0 then
  |   | σ_i ← UniformRandom({−1, +1})
  | end
  | V ← V \ i
end
```

Firstly, the approach classifies the nodes into trapped and swing nodes. This is done by comparing $|x_{\hat{p},i}|$ with the cutoff $\epsilon$ for all nodes $i = 1, 2, ..., n$. Then, the state $\mathbf{x}_{\hat{p}}$ is binarized by mapping swing nodes to zeros and trapped nodes $\mathbf{x}_{\hat{p},j}$ to $\sigma_j \in \{+1, -1\}$ based on the signs of the state values. This is motivated by the "freeze-out" of trapped nodes, in the sense that their magnitudes are sufficiently large such that further evolution of the system does not change the signs anymore.

Next, the approach targets to decide the remaining variables $x_{\hat{p},i}$, $i \in V_0$, based on the trapped nodes. Swing nodes are randomly sequentially assigned binary values $\{+1, -1\}$ based on the fields due to their neighbors, in accordance to the following two steps.

Firstly, every variable $x_{\hat{p},i}$, $i \in V_0$ is set to $\text{sign}(y_i)$, with $y_i = \sum_{j=1,j\neq i}^{n} G_{ij}\sigma_j$ by checking the stability of the configuration under the spin flip of node $i$ (see Methods section). Secondly, the same stability criterion is applied to all nodes $j = 1, 2, ..., n$, leading to a stabilization of the whole configuration. Values of $\sigma_k$ are flipped to $-\sigma_k$ if the stability criterion $\sigma_k = \text{sign}(y_i)$ is violated. The updating order for both steps is randomized.

## Supplementary Note 8 – Performance analysis of CIM with trapping-and-correction (TAC) approach

In this section we demonstrate the performance of CIM with the proposed trapping-and-correction (TAC) approach on ten Max-cut instances, `g05_100.0`–`g05_100.9` (unweighted graphs with $n = 100$ nodes with edge probability 0.5), from the Biq Mac Library [5] (with known solutions) – complementary to the main text.

It can be observed that the mean Ising energy (over 100 trials) of the proposed approach always outperforms CIM (solid lines) and the top 5% results of the approach is clearly below both average and top 5% results of CIM.

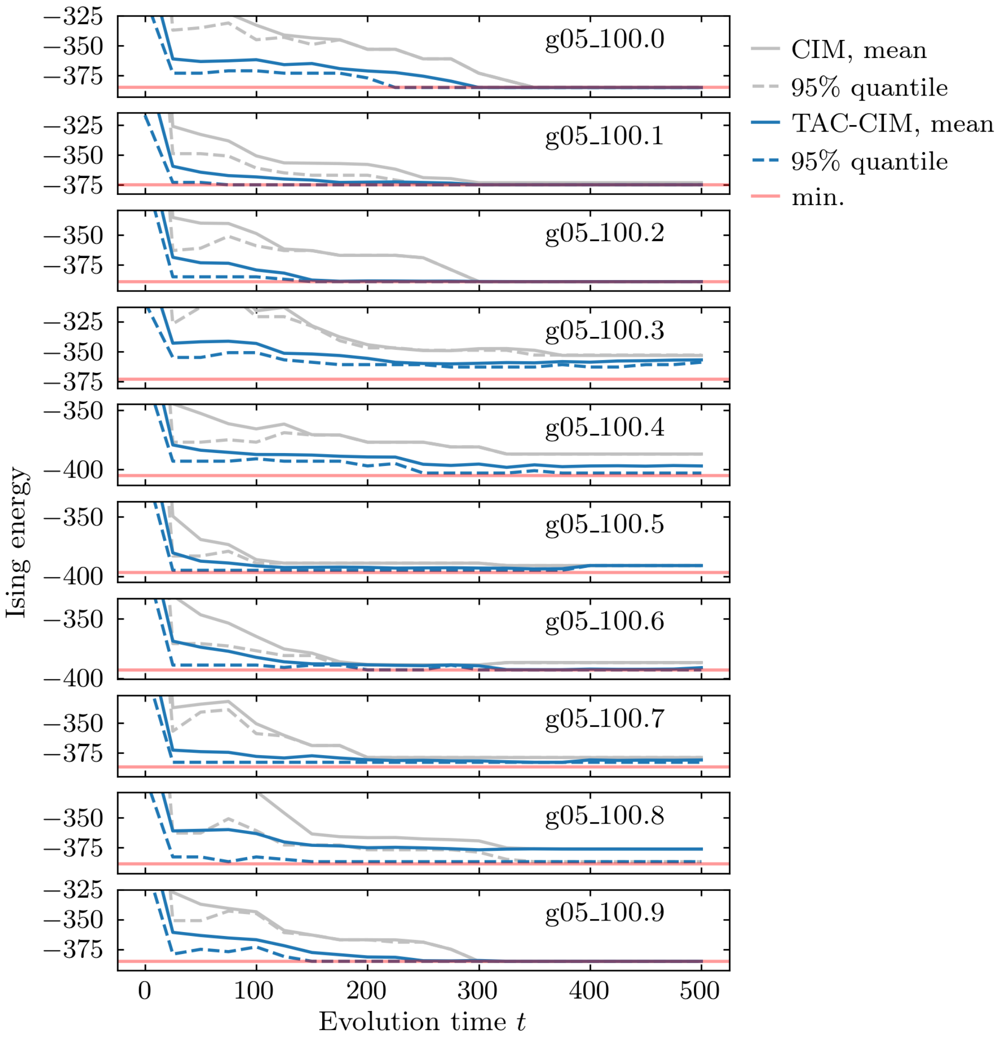


Supplementary Figure 8: The figures compare average (solid lines) and top 5% quantile (dashed lines) Ising energies of ten Max-cut instances with $n = 100$ variables (100 random trials each). The grey lines are standard CIM, the blue lines are CIM with TAC approach. Ground state is marked as "min" and depicted by solid straight line.

## Supplementary Note 9 – Performance analysis of simulated bifurcation and its variants with trapping-and-correction (TAC) approach

In this section, we demonstrate the performance of the TAC approach applied to other general Ising dynamics beyond CIM, such as simulated bifurcation (SB) and its variants, ballistic simulated bifurcation (bSB) and discrete simulated bifurcation (dSB). The size of test graphs, fully connected with random weights ($\pm 1$), are increased to $n = 2000$ to examine possible performance variation in larger problem instances.

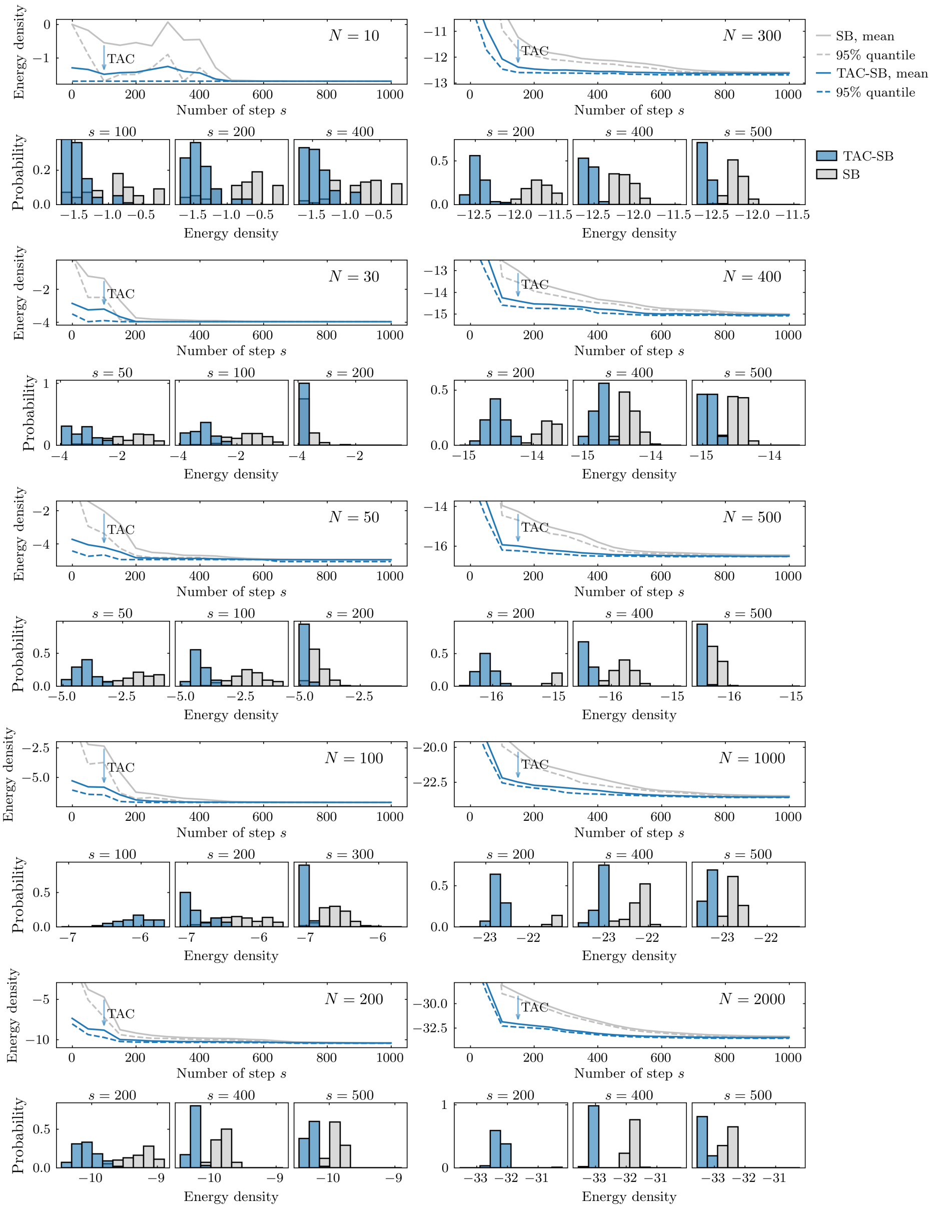


Supplementary Figure 9: Acceleration of simulated bifurcation (SB) through the trapping-and-correction (TAC) approach. Here the Ising energies obtained by the SB algorithm is compared against those by TAC-SB for random graphs of up to $n = 2000$ nodes (the graphs are fully connected with the weight of each edge drawn randomly from $\{-1, +1\}$). The solid curve and the dashed curve indicate the mean and top 5% quantile of the Ising energy respectively. The histograms illustrate the distribution of the obtained Ising energy at different steps.

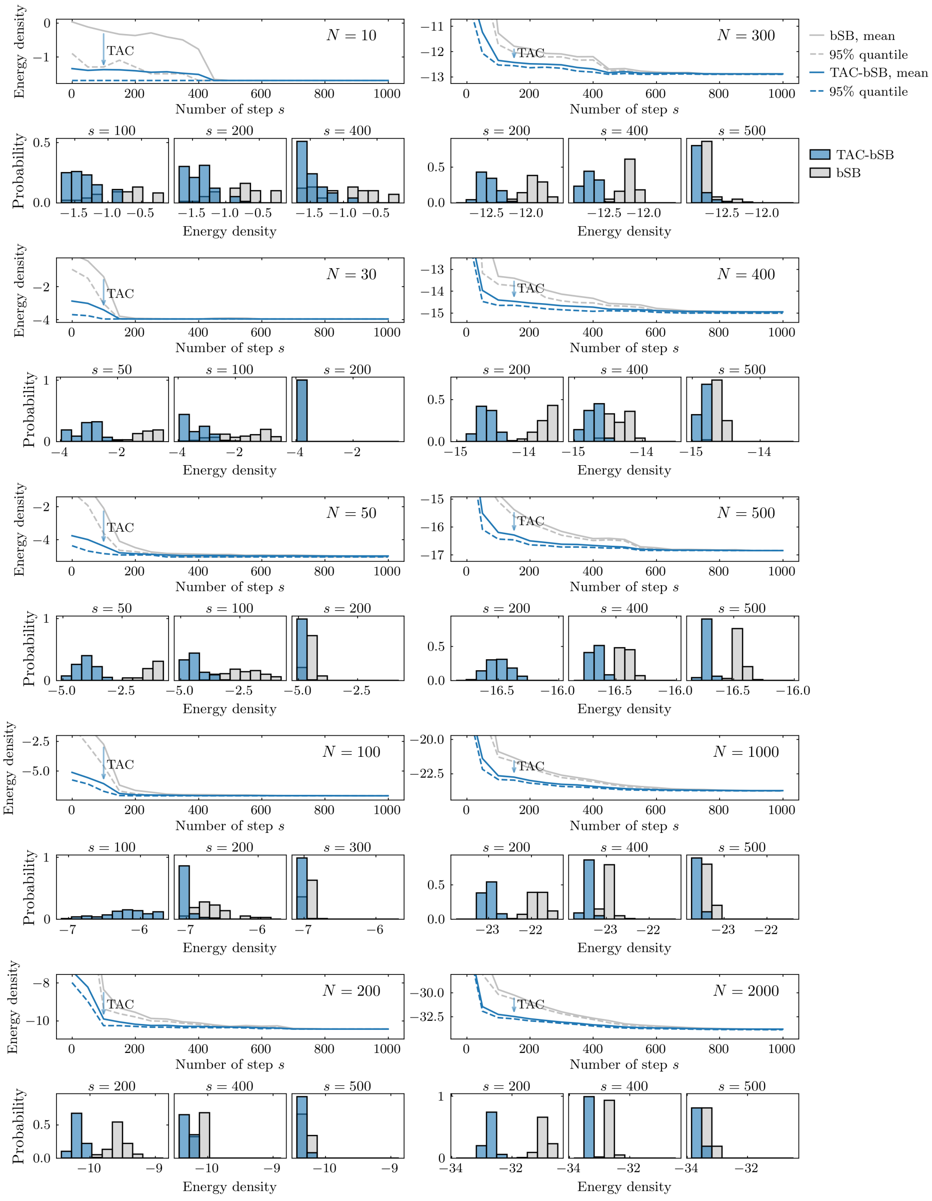

Supplementary Figure 10: Acceleration of ballistic simulated bifurcation (bSB) through the trapping-and-correction (TAC) approach. Here the Ising energies obtained by the SB algorithm is compared against those by TAC-bSB for random graphs of up to $n = 2000$ nodes (the graphs are fully connected with the weight of each edge drawn randomly from $\{-1, +1\}$). The solid curve and the dashed curve indicate the mean and top 5% quantile of the Ising energy respectively. The histograms illustrate the distribution of the obtained Ising energy at different steps.

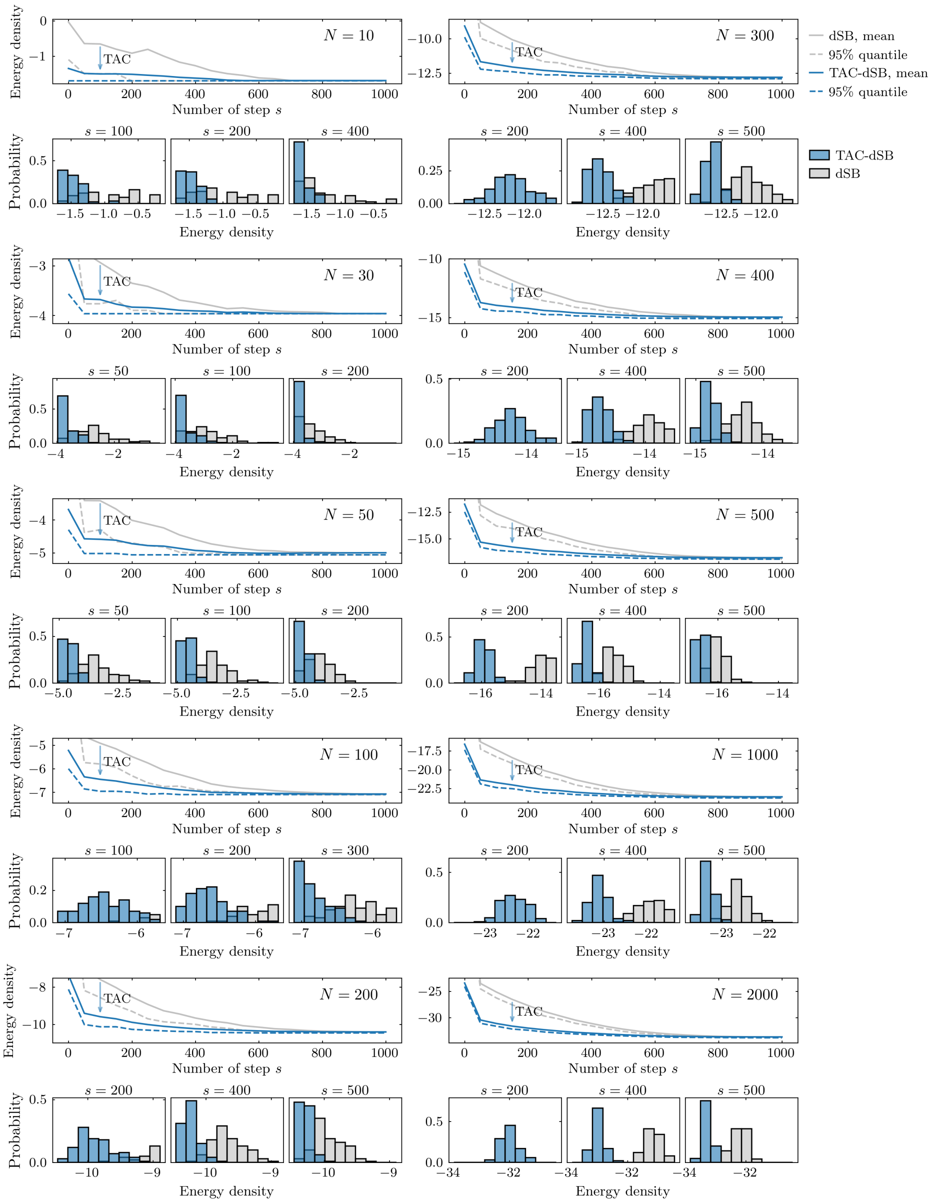


Supplementary Figure 11: Acceleration of discrete simulated bifurcation (dSB) through the trapping-and-correction (TAC) approach. Here the Ising energies obtained by the SB algorithm is compared against those by TAC-dSB for random graphs of up to $n = 2000$ nodes (the graphs are fully connected with the weight of each edge drawn randomly from $\{-1, +1\}$). The solid curve and the dashed curve indicate the mean and top 5% quantile of the Ising energy respectively. The histograms illustrate the distribution of the obtained Ising energy at different steps.

## References


[1] Bredon, G. E. *Topology and geometry*, vol. 139 (Springer Science & Business Media, 2013).

[2] Hartshorne, R. *Algebraic geometry*, vol. 52 (Springer Science & Business Media, 2013).

[3] Golub, G. H. & Van Loan, C. F. *Matrix computations* (Johns Hopkins University Press, Baltimore, MD, 1996).

[4] McMahon, P. L. *et al.* A fully programmable 100-spin coherent ising machine with all-to-all connections. *Science* **354**, 614–617 (2016).

[5] Wiegele, A. Biq Mac Library - a collection of Max-Cut and quadratic 0-1 programming instances of medium size. https://biqmac.aau.at/biqmaclib.pdf (2007).
